# Nonnative Thiol and Aniline Methylation, Including Enantioselective Dithiol Desymmetrization, by Multicomponent Cobamide-Dependent Methyltransferases Using Unactivated Methyl Donors

**DOI:** 10.1002/anie.1537547

**Published:** 2026-09-16

**Authors:** Amardeep Kumar, Osman Ishag Adam, Paras Gupta, Jared C. Lewis

**Affiliations:** Department of Chemistry, Indiana University, Bloomington, Indiana, USA

**Keywords:** B_12_, biocatalysis, cobamide, desymmetrization, methylation

## Abstract

Despite their potential synthetic utility, cobamide-dependent MTases are relatively underutilized for biocatalysis. We developed mixed MtaABC/MtgABC enzyme platforms capable of catalyzing methylation of non-native thiol and aniline substrates using unactivated methyl donors like methanol and glycine betaine. This approach allows highly enantioselective and site-selective methylation of aliphatic thiols with high turnover number (up to 6000), providing the first demonstration of enantioselective non-native catalysis by cobamide-dependent enzymes. The MtaAC+MtgB system was subsequently engineered through site-directed mutagenesis, resulting in large enhancements in activity for the desymmetrization of non-native ester-containing prochiral thiols. These findings establish MTase complexes as a promising platform for non-native biocatalysis.

## Graphical Abstract

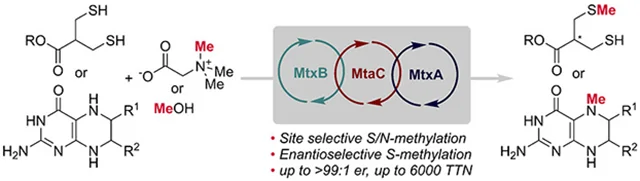

## 1 ∣ Introduction

**FIGURE 1 ∣:**
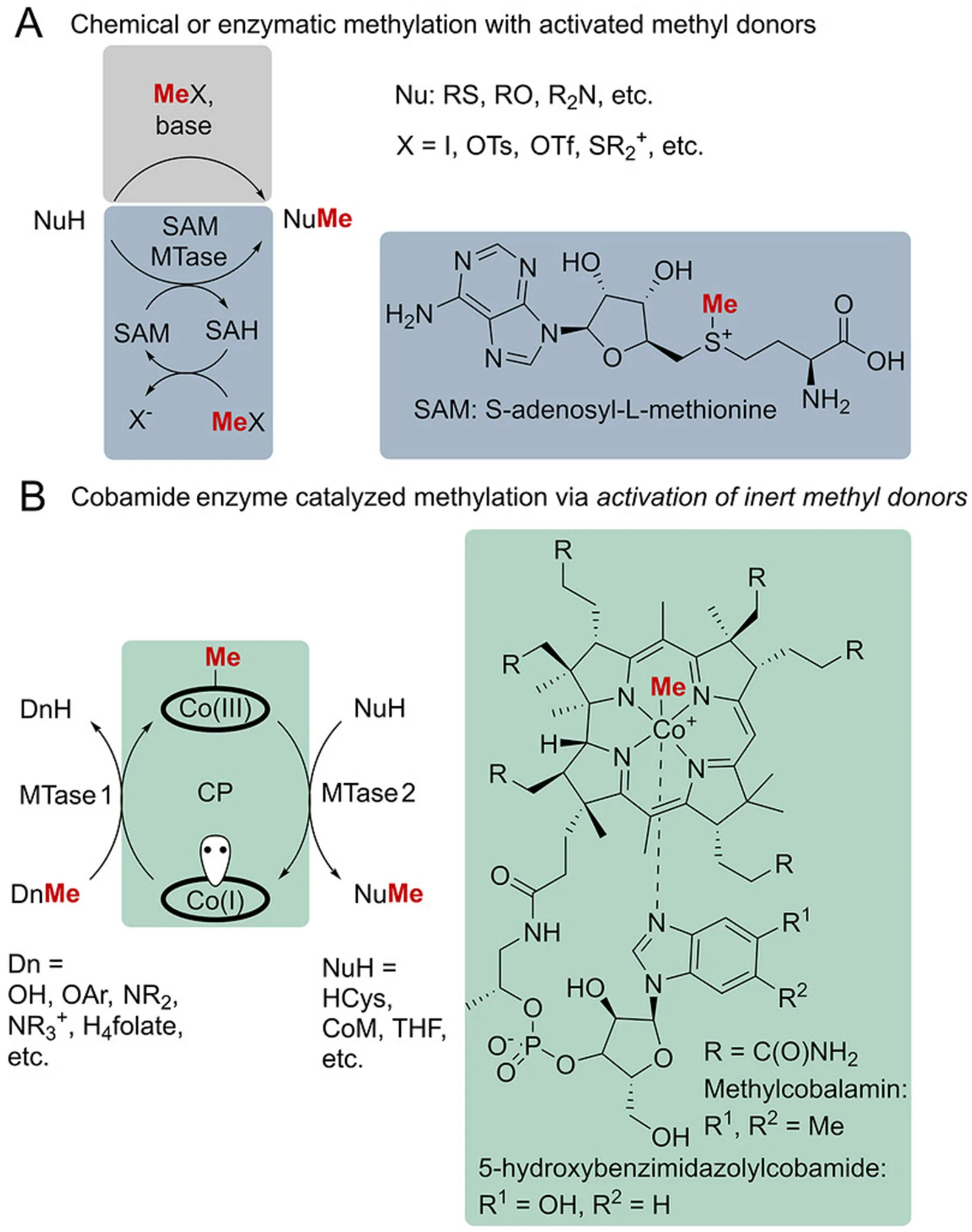
(A) Common chemical [14] and enzymatic [6] methods for heteroatom methylation. (B) General scheme for multi-component 5-hydroxybenzimidazolylcobamide [15] (HbCba)-dependent methyltransferase (MTase) systems comprising MTase1, MTase2, and a corrinoid protein (CP) using different types of unactivated methyl sources [4] for native methylation. Structures of MeCbl and methylated HbCba cofactors are shown (formal charge on Co(III) indicated as superscript).

Methyl groups are present in approximately 70% of pharmaceutical compounds and play a crucial role in enhancing the physical and chemical properties of drug molecules [1]. However, the development of sustainable methods for selective heteroatom methylation has posed significant challenges over the years. Traditional methylation methods rely on activated methyl sources like methyl iodide, methyl triflate, and dimethyl sulfide, combined with excess strong base (Figure 1A) [2]. These methyl donors are highly toxic due to their ability to methylate DNA and other biological molecules, and the excess reagents required to drive methylation to high yields lead to low atom economy and undesirable side reactions. These problems are exacerbated for substrates containing multiple reactive heteroatoms since site- and/or chemoselective methylation is then required [2]. To address these challenges, the development of new, efficient, and sustainable methods for selective methylation is essential [3].

In nature, methyltransferases (MTases) catalyze heteroatom methylation using distinct mechanisms [4], and these are increasingly used for applications in biocatalysis [5, 6]. Perhaps the most well-established MTases are the *S*-adenosyl-L-methionine (SAM)-dependent enzymes that catalyze C-, N-, O-, and S-methylation through a polar (S_N_2) mechanism, producing *S*-adenosyl-homocysteine (SAH) as a byproduct (Figure 1A) [7, 8]. The synthetic utility of SAM and its analogues is limited by its high cost (~$1300 per gram) and instability [9]; however, while these cofactors can be regenerated, the terminal methyl donors in such schemes are the same toxic reagents used in conventional synthetic methylation reactions (methyl iodide [10], methyl toluene sulfonate [11], etc.). A unique member of the cobalamin-dependent radical SAM enzymes, TsrM [12], has also been shown to catalyze heteroatom methylation on nonnative substrates [13]. This enzyme uses SAM to methylate its cob(I)alamin cofactor to generate a methyl cob(III)alamin (Me-Cbl) intermediate, which in turn methylates substrates via a polar mechanism.

A diverse set of cobamide-dependent MTases have been shown to catalyze heteroatom methylation without the need for SAM using a range of methyl donors, including methylated amines/amino acids, anisoles, dichloromethane and even methanol (Figure 1B) [4]. While cobalamin-dependent methionine synthase (MetH) [16] is perhaps the most well-known of these enzymes, a variety of multi-subunit MTases that use 5-hydroxybenzimidazolylcobamide (HbCba) as a cofactor have proven more useful for biocatalysis to date [17]. These enzymes typically comprise two methyltransferases (MTase1/2) and a corrinoid protein (CP) [4]. MTase1 binds and activates a specific methyl donor and transfers the methyl group to the CP. MTase2 then transfers the methyl group from the CP to a specific acceptor substrate. Notably, these enzymes have been reconstituted with cobalamins [18–22] (e.g., Me-Cbl ~$15/g), which are more readily available from commercial sources than HbCba and enable enzymatic cofactor regeneration using low-cost and/or environmentally benign methyl donors.

The Kroutil group has explored the use of HbCba-dependent MTases for reversible and selective methylation and demethylation of aromatic ethers [20, 23, 24]. An early study showed that dhaf4610 (MTase1) from *Desulfitobacterium hafniense* [25], coupled with the CP dhaf4611 reconstituted with MeCbl, enables reversible methyl transfer of catechol-based substrates, achieving high conversions for demethylation and good regioselectivity for methylation without the need for a second MTase [23]. A subsequent effort demonstrated that the MTaseI veratrol-O-demethylase (vdmB) from *Anaeromyxobacter dehalogenans* in combination with dhaf4611 catalyzes regioselective demethylation of methyl phenyl ethers, reaching 77% conversion with >99% regioselectivity for 3,4-dimethoxyphenol and broad substrate tolerance, including natural products [24]. To address equilibrium limitations, a recent study showed that thiols can be used as methyl acceptors with dhaf4610+dhaf4611 to achieve >90% conversion of guaiacol derivatives, enabling gram-scale synthesis of hydroxytyrosol with 97% yield [17]. These advances establish cobamide-dependent enzymes as versatile, green biocatalysts for selective ether cleavage in anisoles and methylation of phenols.

We envisioned that using both MTases involved in cobamide-dependent MTase complexes would allow the use of simpler and more readily available methyl donors and significantly expand the synthetic utility of these enzymes for biocatalytic heteroatom methylation. Toward this end, MtaABC from *Methanosarcina barkeri* caught our attention since it catalyzes methylation of 2-mercaptoethanesulfonate (coenzyme-M, CoM) using methanol as the methyl donor and generates water as a byproduct [26]. Like-wise, we were interested in MtgABC from *D. hafniense* due to its ability to methylate tetrahydrofolate (THF) using glycine betaine (GB), a natural food supplement, generating dimethyl glycine as a water-soluble byproduct that could be readily removed from the methylated product [22, 27, 28]. In both systems, collectively MtxABC (where Mtx: Mta or Mtg), MtxA is the MTase2 that activates the methyl acceptors (CoM or THF), MtxB is the MTase1 that activates the methyl donors (MeOH or GB), and MtxC is the CP, which natively binds HbCba (Figure 1B). Despite extensive studies on the mechanisms and native reactivity of these enzymes [22, 26, 29], their use in non-native methylation biocatalysis has not been reported.

In this study, we establish that these enzymes can methylate a broad range of non-native thiols and anilines in good conversion. We further show a mix-and-match strategy [24], in which different MTase1 enzymes are used with a given MTase2 enzyme to achieve excellent conversion for thiol and aniline methylation. Examples of site- and stereoselective methylation were achieved, and turnover numbers up to 6000 were obtained for some substrates. Furthermore, targeted active-site mutagenesis of MtaA enabled enhanced activity toward nonnative ester substrates, facilitating enantioselective desymmetrization and expanded substrate scope. These latter findings constitute the first examples of non-native enantioselective catalysis by an HbCba-dependent enzyme. This work expands the synthetic utility of HbCba-dependent methyltransferases beyond their native functions and demonstrates how HbCba-dependent proteins can serve as chiral environments to control enantioselectivity of non-native transformations.

## 2 ∣ Results and Discussion

We previously showed that several CPs, including MtaC, dhaf4611, TCP, and MMCP, all reconstituted with HOCbl, catalyze selective *N*-alkylation of anilines using diazo esters as alkylating agents [21]. Of these enzymes, MtaC showed the highest activity, selectivity, and rate acceleration for this non-native chemistry, and it could be produced in high yield. MtaA expressed similarly well, but MtaB was expressed as inclusion bodies and had to be renatured using urea treatment followed by dialysis (see Supporting Information, Section II.E) [29]. MtgA and MtgB were readily expressed, but MtgC was not, a deficiency noted by others [22]. While MtaC and MtgC only have 40% identity, we nonetheless hoped that MtaC could be used in place of MtgC. The MTases were all purified by Ni-NTA affinity chromatography. The native activity of the MtxB enzymes toward HOCbl was validated using their native methyl donors, and the ability of the MtxA enzymes to methylate their native acceptors using MeCbl was likewise confirmed using previously reported methods [29, 30].

**FIGURE 2 ∣:**
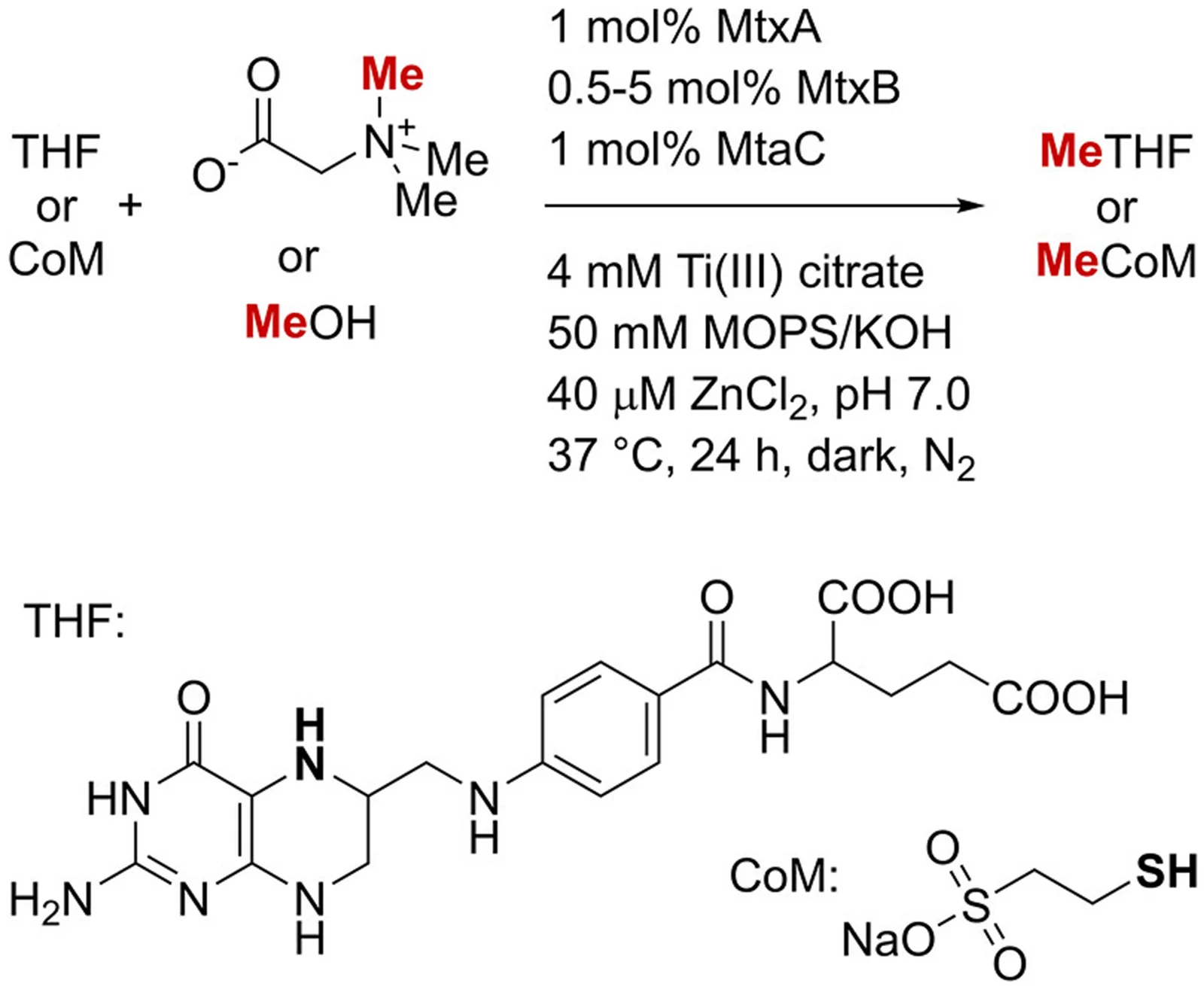
Methylation of tetrahydrofolic acid (THF) and coenzyme M (CoM) catalyzed by mixed MtxABC systems, with the site of methylation shown in bold.

Conditions were next optimized for the native MtxABC reactions using MtaC in place of MtgC and using MeCbl in place of HbCba (Figure 2) [22]. Despite these substitutions, MtgAB+MtaC catalyzed selective N5-methylation of tetrahydrofolate (THF), the native substrate for MtgABC, with quantitative conversion using a two-fold excess of GB as methyl donor (Table 1, Entry 1). As expected, omitting MtgA, MtgB, or MtaC eliminated substrate conversion (Table 1, Entry 2), showing that each subunit is required for catalysis using GB as a methyl donor. Methylation of THF using stoichiometric MeCbl was also examined. In the presence of MtgA and MtaC, good conversion to the expected product was observed (Table 1, Entry 3), confirming that the ternary enzyme complex is not required for substrate methylation if MeCbl is provided [18, 22]. No background reaction was observed in the absence of these enzymes (Table 1, Entry 4), showing their importance for substrate activation to enable methylation by MeCbl [31].

Methylation of CoM by Mta enzymes using MeOH as a methyl donor was also investigated. Preliminary reactions conducted using the same conditions as above led to only 12% ± 3% conversion, but optimizing concentrations of Ti(III) citrate, ZnCl_2_, CoM, and MeOH improved this yield up to 40%. Similar to THF methylation, CoM methylation by MeOH requires MtaA, MtaB, and MtaC (Table 1, Entries 5/6), but only MtaA and MtaC are required for methylation when stoichiometric MeCbl is provided (Table 1, Entries 7/8). Given the relatively low yield for methylation of CoM using MeOH as a methyl donor, we examined whether MtaA could be used in combination with MtgB and MtaC. Indeed, quantitative conversion to the desired product could be obtained under these conditions, showing how the superior methyl activation by MtgB can be interfaced with the thiol activation of MtaA to improve overall biocatalytic efficiency (Table 1, Entry 9).

**FIGURE 3 ∣:**
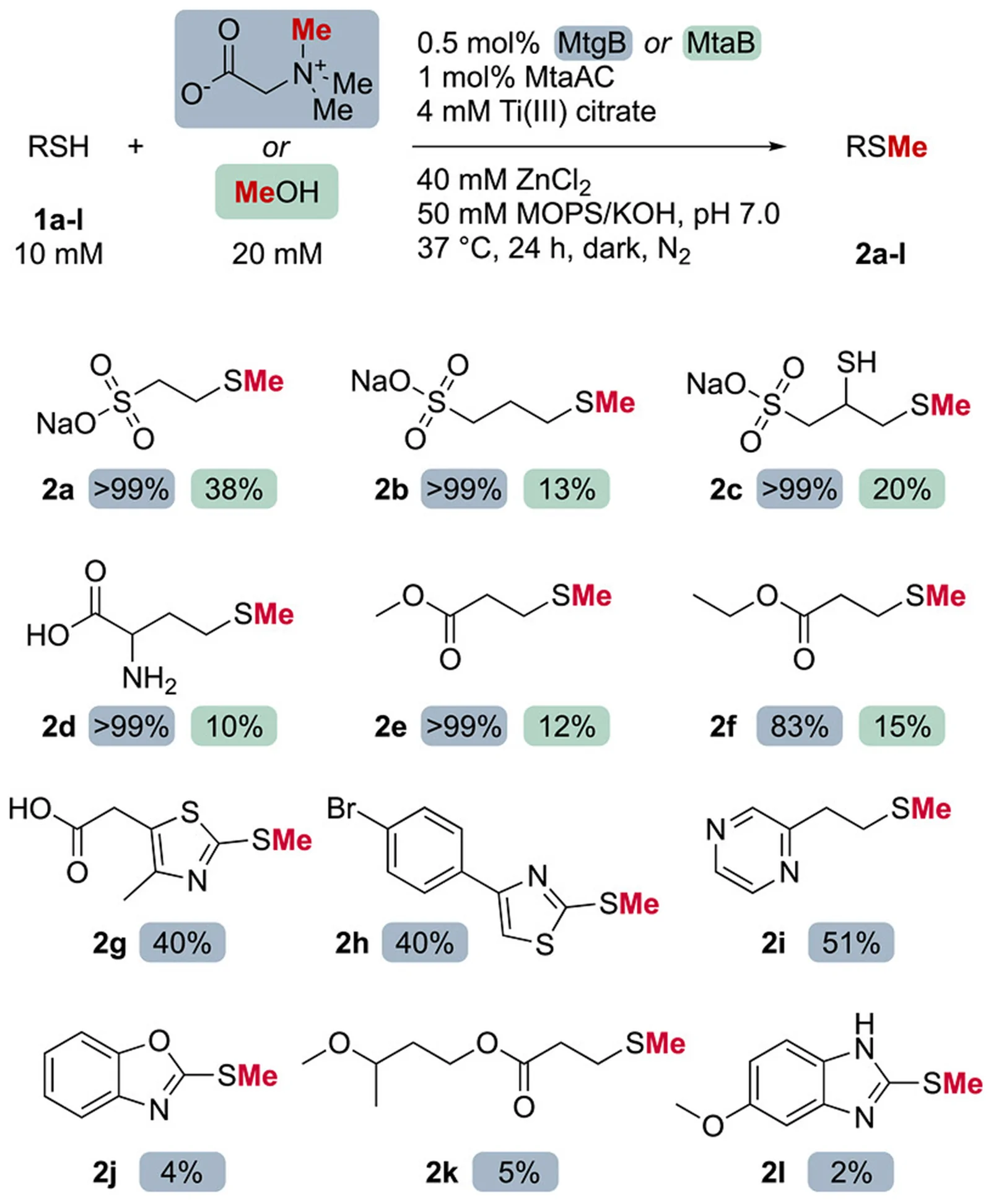
Scheme and substrate scope for non-native *S*-methylation using MtxABC system. Reaction condition includes 10 mM of thiol (1a-i), 20 mM glycine betaine or 5%v/v MeOH as methyl source, 0.5 mol% MtgB or MtaB, 1 mol% MtaA, 1 mol% MtaC, 4 mM Ti(III) Citrate, 40 μM ZnCl_2_, 50 mM MOPS/KOH, pH 7.0, 37°C, 24 h, N_2_ atmosphere. Conversions in blue boxes are for reactions catalyzed by MtgB using GB as a methyl donor, while conversions in green boxes are for reactions catalyzed by MtaB using methanol as a methyl donor (see Table S15).

Given the efficiency of CoM methylation using MtaAC in combination with MtaB or MtgB, we next explored the substrate scope of these systems (Figure 3). Several thiols were methylated in excellent conversion using GB as a methyl donor and in modest conversion using MeOH as a methyl donor. Aliphatic thiols (**1a-f**) having shorter side chains were preferred, and several functional groups, including sulphonic acids, carboxylic acids, amines, and esters, were tolerated. High site selectivity (>100:1) was observed for substrate **1c**, where the enzyme showed a strong preference for the terminal thiol over the internal thiol (see Supporting Information, Section XVII.B). Methylation of **1c** using one equivalent of MeI afforded a 50:50 mixture of mono- and dimethylated products, and two equivalents led to >100:1 selectivity for dimethylation (see supporting information section XII). Preparative-scale (0.3 mmol) reactions were carried out to isolate products **2e** and **2f**, and TON values up to 3000 were observed. Mixed results were obtained for bulkier substrates. For example, thiazole-substituted thiols **1 g**and **1 h** and pyrazine-substituted ethanethiol **1i** were methylated in moderate yield, but benzazoles **1j** and **1l** and the more extended **1k** all gave poor conversion. Unfortunately, even the modest yields of MtaB for methylation of CoM did not translate to other substrates, and low conversions were observed in all cases using MeOH as a methyl donor. Nonetheless, promising substrate tolerance was observed, suggesting that directed evolution could be used to improve the efficiency of this system.

**FIGURE 4 ∣:**
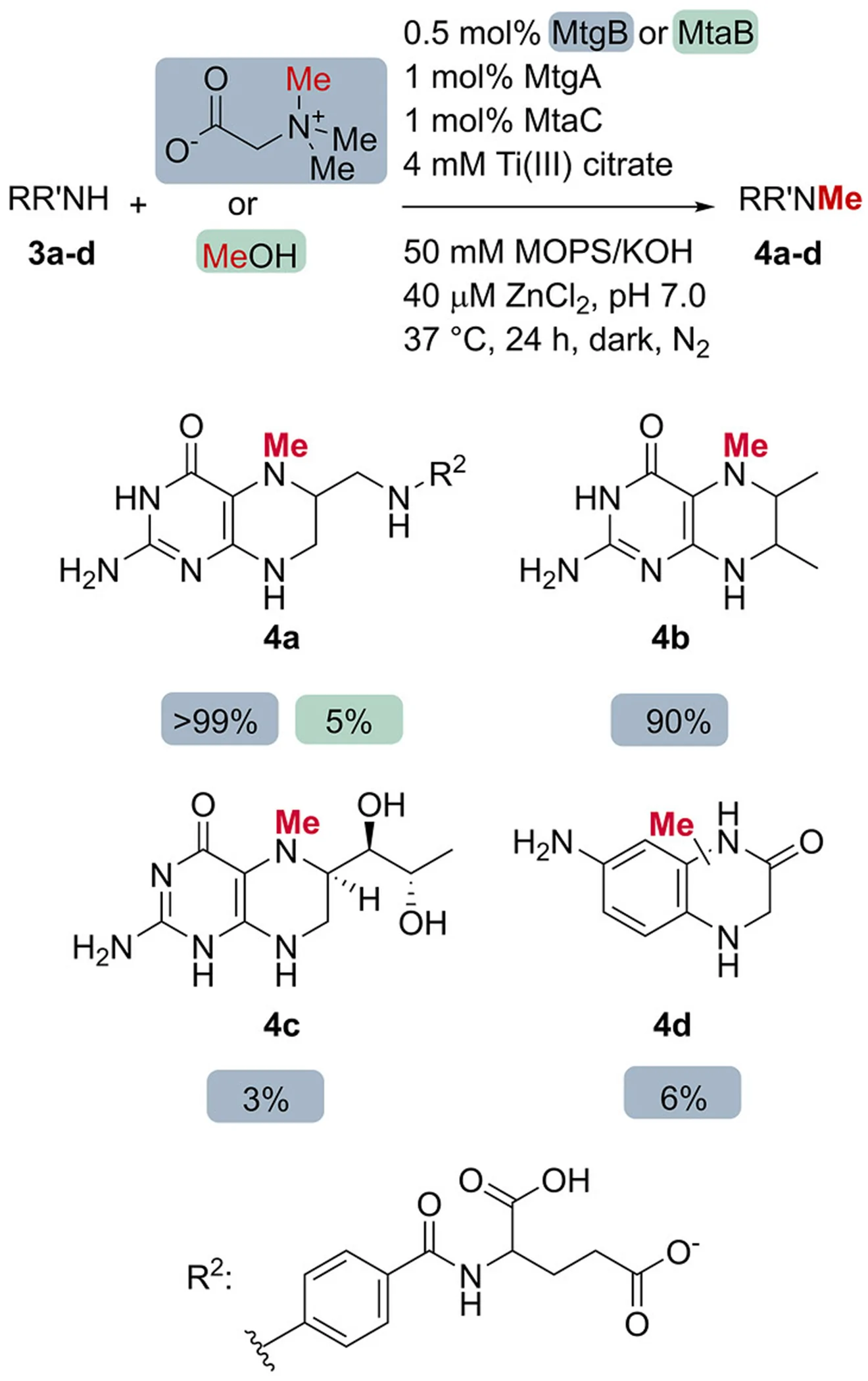
Substrate scope for methylation by MtgA+MtxB+MtaC. Reaction condition includes 10 mM of aniline (**3a**–**d**), 20 mM glycine betaine or 5%v/v MeOH as methyl source, 0.5 mol% MtgB or 5 mol% MtaB, 1 mol% MtaA, 1 mol% MtaC, 4 mM Ti(III) Citrate, 40 μM ZnCl_2_, 50 mM MOPS/KOH, pH 7.0, 10% DMSO as co-solvent for **3b–d**, 37°C, 24 h, total reaction volume 400 μL, N_2_ atmosphere. Conversions in blue boxes are for reactions catalyzed by MtgB using GB as a methyl donor, while conversions in green boxes are for reactions catalyzed by MtaB using methanol as a methyl donor (see Table S15).

Given that MtaA accepted a diverse set of thiols as substrates, we hypothesized that MtgA could be similarly used for non-native aniline methylation. Unfortunately, however, while a truncated derivative of the native substrate was methylated in good yield to give **4b**, related compounds **4c** and **4d** were formed in low yields (Figure 4). No conversion was observed for simple substituted anilines and tetrahydroquinoxaline, showing that more extensive substrate recognition by MtgA is required for *N*-methylation compared to *S*-methylation catalyzed by MtaA. Interestingly, THF could be methylated using MeOH as a methyl donor using MtaB, but low conversion was observed.

**FIGURE 5 ∣:**
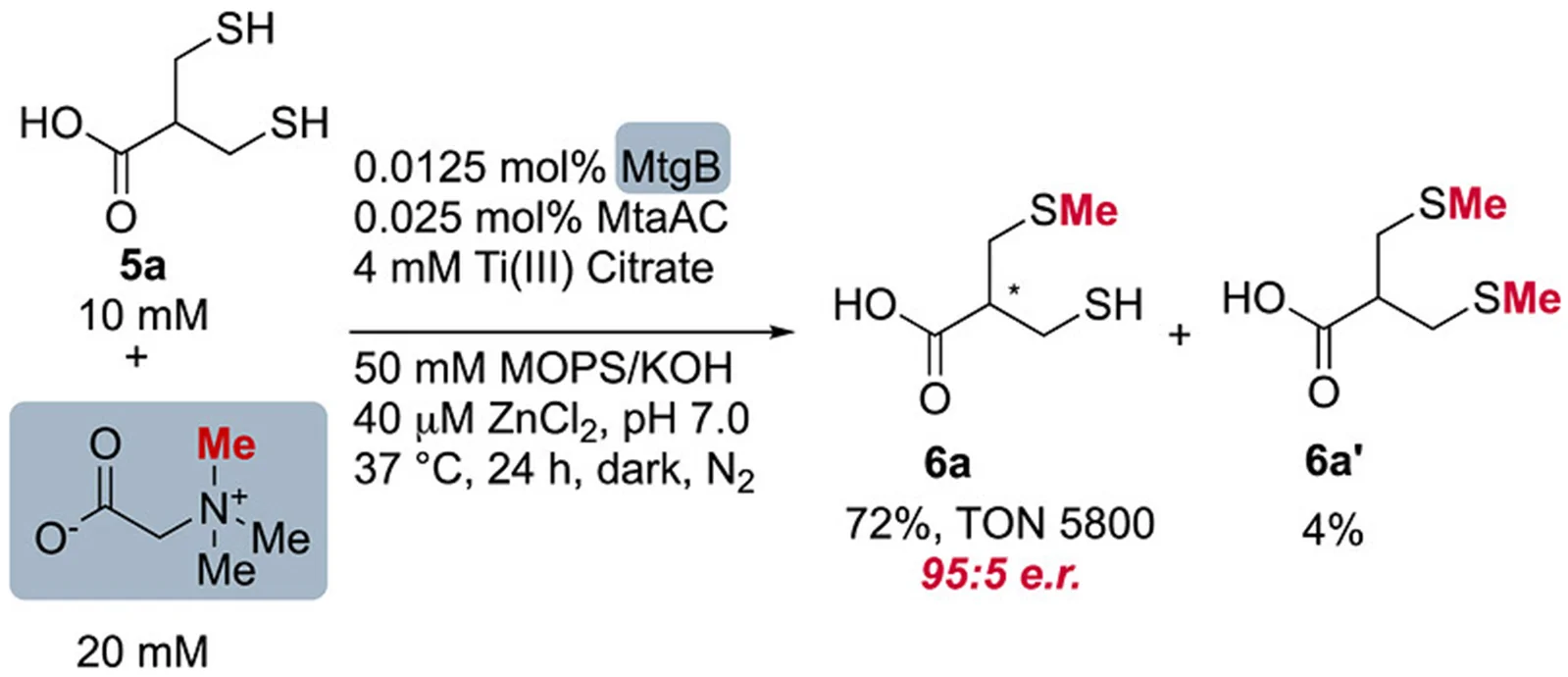
Scheme for enantioselective S-methylation of symmetric dithiol (**5a**) catalyzed by MtaAC+MtgB. Optimized reaction condition include 10 mM **5a**, 20 mM glycine betaine, 0.0125 mol% MtgB, 0.025 mol% MtaA, 0.025 mol% MtaC, 4 mM Ti(III) Citrate, 50 mM MOPS/KOH, pH 7.0, 40 μM ZnCl_2_, 37°C, 24 h, N_2_ atmosphere. Yield for **6a** was determined by isolating product from semi-preparative-scale (0.3 mmol) bioconversion reaction; conversion to **6a’** was determined by LC–MS analysis (see Table S15).

The absence of a background reaction between MeCbl and THF or CoM and the requirement for MtaC+MtxA shows that these enzymes accelerate methylation via a combination of substrate and/or MeCbl activation. We therefore wondered whether this catalyst control could be extended to stereoselective methylation. Specifically, we envisioned that prochiral substrate 3-mercapto-2-(mercaptomethyl) propanoic acid (**5a**) could be desymmetrized via enantioselective methylation. Previously optimized conditions provided monomethylated (**6a**) and dimethylated (**6a’**) products in a 60:40 ratio. Reducing catalyst loading to 0.0125 mol% MtgB and 0.025 mol% MtaAC and monitoring the reaction established that 72% conversion to monomethylated product (**6a**) and 4% of dimethylated product (**6a’**) could be obtained. A preparative-scale (0.3 mmol) bioconversion reaction was then performed, and monomethylated product **6a** was isolated in 52% yield, corresponding to a TON of up to 5800 (Figure 5). This high TON was achieved despite the modest *K*_M_ (0.89 ± 0.02 mM) and *k*_cat_ (2.2 ± 0.01 min^−1^) values obtained from steady-state kinetic analysis of desymmetrization of **5a**, highlighting the robustness of MtgB+MtaAC for biocatalysis. Most notable, however, is the high enantioselectivity (95:5 enantiomeric ratio) observed for the product, given that no previous studies have reported highly enantioselective non-native catalysis by a cobamide-dependent protein.

To gain structural insight into the enantioselectivity of methylation by MtaAC, AlphaFold3 (AF3) was used to generate models of a complex comprising MtaA, MtaC, **5a**, Zn^2+^, and MeCbl (Supporting Information, Section IV). The majority of the models, including all top-ranked models, showed the *pro*-R thiol proximal to bound Zn^2+^ and the MeCbl methyl group, analogous to the binding orientation of CoM in the crystal structure of the MtaA/CoM/Zn^2+^/MeCbl complex [31]. The *R*-configuration of **6a** was experimentally confirmed by x-ray crystallography following methylation of the carboxylic acid using TMS diazomethane, thiol alkylation with *p*-bromobenzyl bromide, and oxidation of the thioethers to give the corresponding disulfone (Supporting Information, Section V). Consistency between the stereochemical outcome of MtaAC-catalyzed methylation of **5a** from modeling and x-ray crystallography provided support for use of AF3 models of MtaAC in further engineering efforts.

**FIGURE 6 ∣:**
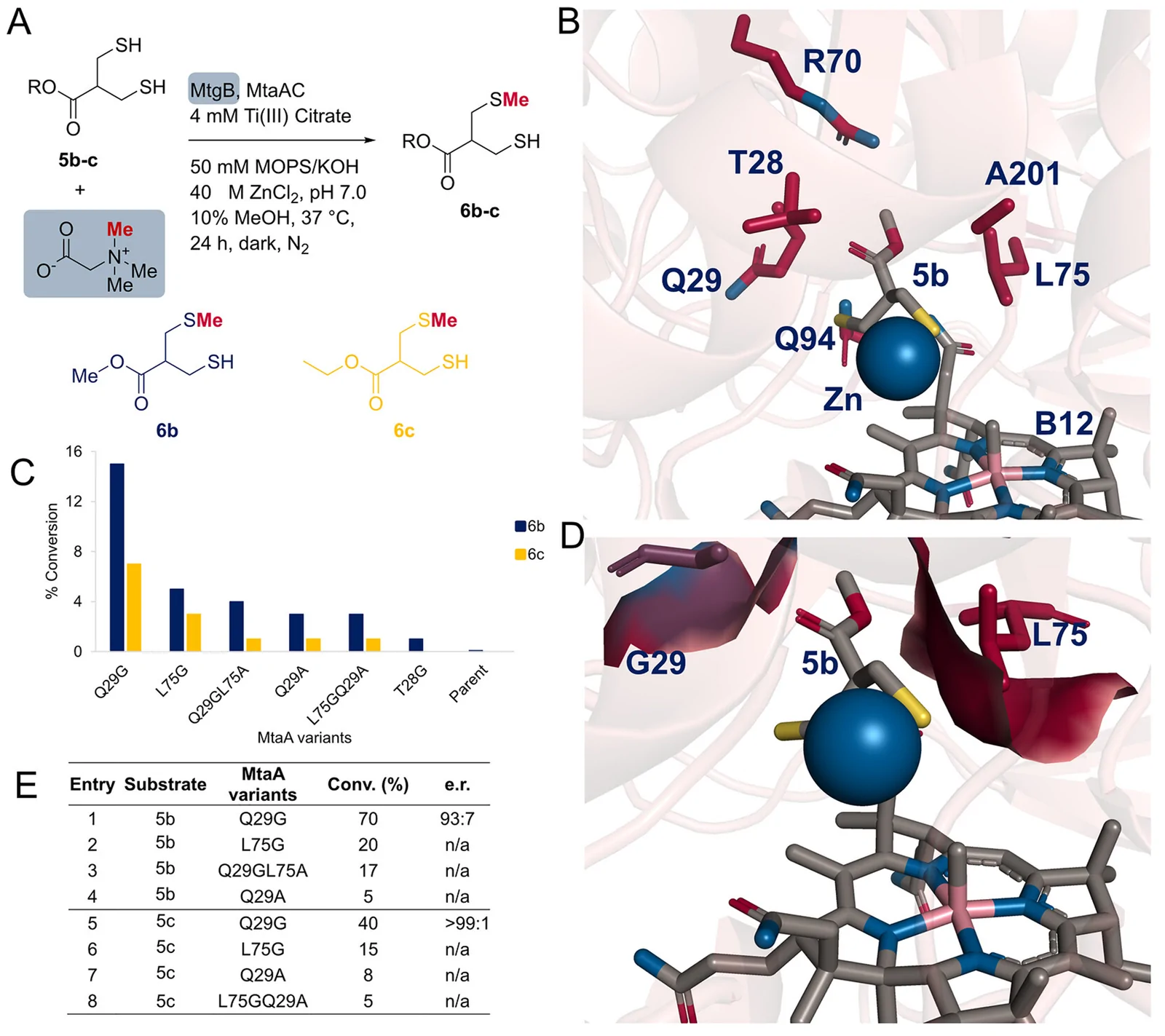
(A) Scheme and substrate scope for thiol desymmetrization. (B) AF3 model of a complex of MtaA, MtaC, B_12_, Zn(II), and **5b** used to select residues for mutagenesis (red). (C) Plot showing conversion to dithiol **5b-c** desymmetrization using purified MtaA variants; reaction condition include 10 mM **5b-c**, 20 mM glycine betaine, 0.0125 mol% MtgB, 0.025 mol% MtaA variant, 0.025 mol% MtaC, 4 mM Ti(III) Citrate, 50 mM MOPS/KOH, pH 7.0, 10% MeOH as co-solvent, 40 μM ZnCl_2_, 37°C, 24 h, N_2_ atmosphere. (D) AF3 docking model of substrate **5b** bound in the active site of the engineered MtaA Q29G variant. The substrate is positioned between Gly29 (purple surface) and Leu75 (crimson surface). (E) Table showing conversion and enantioselectivity for **5b** and **5c** methylation using the same conditions as (c), except 1 mM **5b-c**, 1.0 mol% MtgB, 2.0 mol% MtaA variants, 2.0 mol% MtaC. Enantiomeric ratios for entries 2–4 and 6–8 were not provided due to low product signal in the UHPLC chromatogram at 210 nm. Yield for **6a-b** was determined by isolating product from a preparative-scale (0.3 mmol) bioconversion reaction; conversion to **6c** was determined by GC–MS analysis of triplicate reactions (see Table S15).

Low conversion was observed for methyl ester (**5b**, <1%) and ethyl ester (**5c**, none detected) derivatives of carboxylic acid **5a** (Figure 6A) despite the fact that methyl and ethyl ester substrates with a single thiol group were tolerated (Figure 3). This finding suggested that the added bulk of dithiols **5b** and **5c** was responsible for their poor reactivity and that mutagenesis aimed at expanding the active site cavity could correct this issue. An AF3 model of a complex comprising MtaA, MtaC, Zn^2+^, methyl cobalamin, and **5b** in place of **5a** suggested six active-site residues (Q29, L75, A201, T28, Q94, and R70) as promising candidates for mutagenesis (Figure 6B). Each residue was individually mutated to alanine or glycine to reduce steric bulk, and the resulting variants were screened for methylation activity on **5b** and **5c**. Notably, several beneficial mutations were identified: Q29G exhibited a 15-fold increase in monomethylation activity toward **5b** relative to the parent enzyme, while L75G and Q29G+L75A displayed 3–6-fold improvements (Figure 6C). Furthermore, Q29G enabled detectable monomethylation of **5c** (7% conversion), and L75G afforded 3% conversion, whereas no activity was observed with wild-type MtaA. Upon further optimizing co-solvent and reaction conditions, Q29G provided up to 70% conversion to **6b** with a 93:7 enantiomeric ratio and 40% conversion to **6c** with >99:1 enantiomeric ratio (Figure 6E).

Analysis of the AF3 model for the **5b** complex with MtaA Q29G, MtaC, Zn^2+^, and MeCbl suggests that substitution of the Q29G mutation creates a narrow cleft for the ester substituent, which clashes with Q29 in the WT enzyme (Figure 6D and Supporting Information, Section III). Active-site pocket analysis revealed that the cavity volume increased from 492 Å^3^ in the parent MtaA to 608 Å^3^ in the Q29G variant to better accommodate ester substituents while still orienting the reactive thiol toward the MeCbl cofactor. Together, these results provide a plausible rationale for the enhanced catalytic activity of the Q29G variant and indicate that further expansion of substrate scope should be possible via directed evolution.

## 3 ∣ Conclusion

This study establishes that multi-component, HbCba-dependent methyltransferases can be repurposed as efficient and versatile biocatalysts for nonnative heteroatom methylation when reconstituted with MeCbl. This capability was uniquely enabled by a “mix-and-match” strategy using MTase1 and MTase2 components from different native systems. While compatibility of Mtase1 enzymes and CPs from different species had been established [24], use of multiple MTase enzymes allowed for efficient methyl donor activation with complementary substrate activation and methyl transfer, resulting in robust methylation activity across a range of thiol and aniline substrates. The mixed MTase systems exhibit good-to-excellent conversions, high site selectivity even in the presence of multiple reactive heteroatoms, and turnover numbers reaching nearly 6000, highlighting their practicality and efficiency. Collectively, these findings establish cobamide-dependent MTases as attractive platforms for sustainable, selective methylation using benign and inexpensive methyl donors.

In addition, we showed that mixed MTase-catalyzed thiol methylation can be extended to enantioselective desymmetrization of prochiral dithiols. This result constitutes the first example of highly enantioselective nonnative catalysis by a cobamide-dependent enzyme. Furthermore, structure-guided mutagenesis of MtaA led to substantial improvements in activity toward bulkier ester substrates, supporting the feasibility of expanding substrate scope through protein engineering. These results indicate that the MTase active site can be tuned to accommodate more complex substrates while maintaining high stereocontrol. As such, this system provides a foundation for the development of engineered cobamide-dependent enzymes capable of imparting both site- and enantioselectivity to B_12_-catalyzed reactions [32].

## Supplementary Material

Supporting Info

Additional supporting information can be found online in the Supporting Information section.

## Figures and Tables

**TABLE 1 ∣:** Methylation efficiency in the presence of different Mtx enzyme combinations.

Entry	Substrate	Me Donor	MtxAB (x)	Variation	Conv. (%)
1^a^	THF	GB	g	+MtgA, +MtgB, and +MtaC	>99
2^a^	THF	GB	g	−MtgA, −MtgB, or −MtaC	0
3^a^	THF	MeCbl	g	+MtgA and +MtaC	>99
4^a^	THF	MeCbl	g	−MtgA, −MtgB, and −MtaC	0
5^b^	CoM	MeOH	a	+MtaA, +MtaB, and +MtaC	38 ± 3
6^b^	CoM	MeOH	a	−MtaA, −MtaB, or −MtaC	0
7^b^	CoM	MeCbl	a	+MtaA and +MtaC	>99
8^b^	CoM	MeCbl	a	−MtaA, −MtaB, and −MtaC	0
9^c^	CoM	GB	MtaA; MtgB	+MtaC	>99

a Optimized reaction condition includes 10 mM THF, 20 mM glycine betaine (GB) or 10 mM MeCbl, 0.5 mol% MtgB, 1 mol% MtgA, 1 mol% MtaC, 4 mM Ti(III) Citrate, 40 μM ZnCl_2_, 50 mM MOPS/KOH, pH 7.0, 37°C, 24h, N_2_ atmosphere.

b As in (a) except 10 mM CoM, 5% v/v MeOH, 5 mol% MtaB, 1 mol% MtaA, 2 mM Ti(III) Citrate, 2 mM ZnCl_2_.

c As in (a), except 10 mM CoM, 20 mM GB, 1.0 mol% MtaA. Conversion was determined by LC–MS analysis of triplicate reactions. Variation lists enzymes included (+) or omitted (−).

## Data Availability

The data that support the findings of this study are available in the supplementary material of this article
