# Supplementary material for: Nonnative Thiol and Aniline Methylation, Including Enantioselective Dithiol Desymmetrization, by Multicomponent Cobamide-Dependent Methyltransferases Using Unactivated Methyl Donors
Source: Angew Chem Int Ed Engl. Author manuscript; Available in PMC 2026 Sep 29. (PMC13618148; doi:10.1002/anie.1537547)
Supplement: Supplementary file 1 [file NIHMS2211437-supplement-Supporting_Info.docx]

**Non-native Thiol and Aniline Methylation, Including Enantioselective Dithiol Desymmetrization, by Multicomponent Cobamide-Dependent Methyltransferases Using Unactivated Methyl Donors**

Amardeep Kumar^1^, Osman Ishag Adam^1^, Paras Gupta^1^, Jared C. Lewis^1,*^
^1^Department of Chemistry, Indiana University, Bloomington, IN 47405, USA

Table of contents

[I. **Materials and Instruments**	2](#_Toc236693490)

[A) **Materials**	2](#_Toc236693491)

[B) **Instruments**	2](#_Toc236693492)

[C) **Software**	3](#_Toc236693493)

[II. **Enzyme preparation**	3](#_Toc236693494)

[A) **Enzyme gene preparation**	3](#_Toc236693495)

[B) **Mutagensis procedure**	6](#_Toc236693496)

[C) **Transformation**	8](#_Toc236693497)

[D) **Protein Preparation**	8](#_Toc236693498)

[E) **MtaB renaturation procedure**	8](#_Toc236693499)

[F) **MtaA glycine/alanine variants protein preparation**	10](#_Toc236693500)

[G) **ESI-MS spectrum for purified His-tagged proteins**	10](#_Toc236693501)

[III. **AF-3 analysis of engineered variant Q29G**	14](#_Toc236693502)

[IV. **Docking analysis of stereoselectivity**	15](#_Toc236693503)

[V. **X-ray crystallographic determination of the absolute configuration of the 6a**	18](#_Toc236693504)

[VI. **Bioconversion Protocol**	20](#_Toc236693505)

[**A) Analytical scale reaction procedure**	20](#_Toc236693506)

[**B) Semipreparative scale reaction**	21](#_Toc236693507)

[VII. **Native activity measurement and assay development**	21](#_Toc236693508)

[VIII. **Reaction optimization for bioconversion using MeOH and glycine betaine**	23](#_Toc236693509)

[IX. **Reaction optimization and TON for monomethylation**	27](#_Toc236693510)

[X. **Michaelis-Menten kinetics of the desymmetrization of 5a**	28](#_Toc236693511)

[XI. **Turnover number measurement for thiol methylation**	29](#_Toc236693512)

[XII. **Methylation with MeI to measure site selectivity of mono vs demethylation**	29](#_Toc236693513)

[XIII. **Stoichiometric methylation**	30](#_Toc236693514)

[XIV. **Bioconversion analysis for thiol and aniline substrates**	32](#_Toc236693515)

[XV. **LCMS Calibration Curves**	41](#_Toc236693516)

[XVI. **Chiral chromatogram**	44](#_Toc236693517)

[XVII. **Synthetic Procedures**	46](#_Toc236693518)

[XVIII. **References**	67](#_Toc236693519)

I. **Materials and Instruments**

A) **Materials**

Analytical scale bioconversions were carried out in a 1.5 mL plastic eppendorf purchased from VWR Scientific (Radnor, PA). Ti(III) chloride 20% (w/w) in 3% hydrochloric acid was purchased from Thermo Fischer Scientific (Radnor, PA). Ti(III) citrate was prepared by mixing 3.75mL of Titanium(III) chloride 20% (w/w) solution with 50mL of 200 mM sodium citrate solutions. The pH of final solutions was adjusted to 7.5 using sodium carbonate. The concentration of Ti(III) citrate was determined by absorbance at 547nm (Ɛ=100M^-1^cm^-1^). All thiol and aniline substrates were cheap and commercially available and were ordered from Sigma Aldrich (St.Louis, MO) or Ambeed (USA). Luria broth (LB) was bought from Research Products International (Mt. Prospect, IL). Miniprep Kits and Ni-NTA superflow cartridges were purchased from QIAGEN, Inc (Valencia, CA) and used according to the manufacturer’s instructions. Ni-NTA resin was purchased from Fisher Scientific (Hampton, NH). PageRuler™ Plus Prestained Protein Ladder, 10 to 250 kDa (Catalog number: 26619) was purchased from Fischer Scientific (Ipswtich, MA). Primers used for cloning were purchased from Sigma Aldrich (St. Louis, MO). The plasmids harboring the synthesized genes were purchased from Twist Bioscience (South San Francisco, CA).

B) **Instruments**

LC-MS analysis for bioconversions was performed on an Agilent 1200 series system connected to a ZORBAX Eclipse Plus C18 column (2.1 mm x 50 mm, 1.8 μm particle size) with a 1290 Infinity II high-speed pump, a 1260 Infinity II diode array detector, and a 1290 Infinity II multisampler with single-needle configuration connected to a 6135X single quadrupole mass spectrometer with an Agilent Jet Stream ESI source. GC-MS analysis for bioconversions was performed by using Agilent 6890N Network Gas Chromatograph equipped with a module 7683B series autosampler and an Agilent 5975 mass selective detector. Analytical-scale bioconversions were analyzed by GC-MS using a 30m DB5 column (0.25 mm id 0.25 mm DF) from Agilent Technologies (Santa Clara, CA). ^1^H NMR spectra were recorded at room temperature on Varian I400, Varian I500, or Bruker B500 spectrometers. Chemical shifts are reported in ppm from tetramethylsilane with the solvent resonance as the internal standard. Data are reported as follows: chemical shift, multiplicity (s = singlet, d = doublet, t = triplet, q = quartet, br = broad, m = multiplet), coupling constants (Hz), and integration. ^13^C NMR spectra were obtained from the Bruker B500 instruments with complete proton decoupling. Chemical shifts are reported in ppm from tetramethylsilane with the solvent resonance as the internal standard (CDCl_3_: δ77.16 ppm).

C) **Software**

NMR spectra were processed using MestReNova 11.0. GC-MS/LC-MS data were analyzed by MassHunter Software B.09.00 from Agilent Technologies.

II. **Enzyme preparation**

A) **Enzyme gene preparation**

**MtaA/MtaB/MtaC/MtgA/MtgB/MtgC design.**

The MtaABC from *Methanosarcina barkeri* was obtained from The Universal Protein Resource (Uniprot ID: Q46EH4). The sequence of His_6_ was added to the N-terminus of the each sequence. The DhaF4611 from *Methanosarcina barkeri* was obtained from the paper Grimm, 2020^1^. MtgA/MtgB/MtgC genes from *Desulfitobacterium hafniense* (strain Y51) were obtained from uniport. The sequences were optimized, synthesized, and subcloned into pET28a^+^ (except MtaB which was subcloned into pET16b) by *Twist Bioscience* for *E.Coli* expression. The entire DNA sequences of each protein are shown below:

**MtaA** (Uniprot ID: Q48927)

**ATG**TCTGAGTTCACGCTGAAAACACGCCTGCTGGCTGCGCTCGAAGGGAAACCAGTTGATAAAGTTCCTGTATGCAGTGTCACGCAGACCGGCATCGTAGAACTCATGGATAAAGTGGGTGCGGCCTGGCCTGAAGCCCACACAAACCCGGAACTGATGGCCAAACTGGCCATTGCTAACTACGAACTGTCAGGCCTGGAAGCGGTGCGTCTGCCATATTGTCTGACTGTATTAGGCGAAGCTATGGGGTGCGAGATTAATATGGGTACGAAAAATCGCCAGCCGAGCGTGACCGCTTCCCCGTACCCTAAGAATTTAGATGGCGCGGTGGTTCCGGCGGATCTTCTGCAGCGCAATCGCATTCCGGCCGTTCTTGAAGCTATTAAGATCGTGCGCGAGAAGGTTGGTCCGGATGTTCCAATCATCGGGGGAATGGAGGGGCCCGTCACCCTCGCATCAGATCTTATTAGCGTAAAAAGTTTCATGAAATGGAGCATTAAAAAAACCGATTTGTTCGAGCAGGCGCTGGATATTTCGGCGGAAGCGGCGATCGCATACGCGAACGCCATGGTTGAAGCAGGTGCCGATGTTATCGCCATCGCAGACCCTGTAGCCTCGCCCGACTTGATGAGCCCGGAAACTTTCAAGCAATTCCTGCAGAGCCGTTTGCAGAAGTTTTCTGCGGGCGTTAACTCAGTGACCGTGCTTCACATCTGTGGCAAAGTTAACGCGATTCTGAGCGATATGGCAGACTGCGGCTTTGAAGGACTGTCTGTCGAAGAGAAGATCGGTACCGCTGCGGAGGGTAAGAAAATTATCGGCGATCGCGCGCGTCTGGTTGGTAATATTTCTAGCCCATTTACCCTGCTCCCAGGACCTATCGACAAGATTAAAGCTGAAGCGAAAGTGGCGTTAGAAGGAGGCATCGACGTACTTGCCCCGGGGTGCGGCATCGCGCCAATGACCCCGCTGGAAAATGTGAAAGCACTGGTTGCCGCACGCGATGAGTATTACGCC**TGA**

**MtaB** (Uniprot ID: Q46EH3)

**ATG**GCAGCAAAACGCTATACAAGCATGGCCTATGCGAATGCCGATGAAATGACATTCGGCGTGTCAAAGTATCCCGTAAAAGCTGGATTAGATTTAGAGATTGGAGCTGGATATACGATACCGGAAATAAATTATGCGCCGCGTCCAGAGGCAGGCGCTAGTAAGGAAAAATTGATCAAGGAATATGAACGTATTACGACTGATGTCATGGAACGCATGGTGCAGGTGGGGTTTCCCGCCATTATACTGGAAACAGAGCATGTGCAACAGATGTCAAACAACCCAAGCTGGGGCGCTGAAGTGGCTCACGCTCAAAAAACTATAATGGAAAAATATCATGATGAGTACGGTATAAAGTGTGCACTGCGCCATACAATAGGTGATATTCGTGAGAATCGTGAATTTTTGCAGCTTCGTGGGGATAAATATAGCGTGTTTCTGGAGGCTTTTGAGCAGTGTGCCGAGAACGGTGCTGATCTCTTGAGTGTGGAATCTATGGGGGGTAAAGAAGTGTTTGATTATGCGGTCCTTCGCAATGATATACCGGGTTTATTGTATAGTATTGGTTGCCTGGGCTCGATCGATATGGAATTAATCTGGACTGATATTAGTAAGATAGCTAAAAAAACCGGAACCATTTCTGCAGGCGATACCGACTGCGCTCAGGCCAATACTGCAATGTTCATCGGGGGAGGGCTCCTGAACAAAAACCTGGCACATACGATTGCAGTTATCGCTCGAGCGATTTCAGCACCAAGAAGCTTAGTTGCCTATGAGGCTGGTGCCGTGGGGCCTGGCAAAGACTGTGGGTATGAAAATATCATCGTGAAGGCTATAACCGGCATGCCGATGACCATGGAAGGGAAAACCTCTACCTGCGCTCATAGCGATGTAATGGGGAACCTGGTTATGCAATGCTGCGATTGCTGGTCGAATGAATCAGTGGAGTATCATGGAGAATTCGGGGGTACCACGGTCCAATGTTGGAGCGAAACACTCGCCTACGACTGCGCATTAATGAATACAGCTCTGGAAACTAAGAATGACAAAGTGCTCCGCGACTTAATGATGTTGTCGGATCGTTACCGTGACCCCCAGGCATATATGCTGGCTTATGATAATGCTTATCGCGTGGGACAATCAATCGTAAAAGATGGAGACAACATTTATTTACGTGCCAAAAATGCAGCCATCGAGTGCTGTAACATTATAGAAGAAGGTGCCGCTGGGAAACTTGAATTATCTCGGTTCGAAACCAAGGCCCTCGCTGACGCGAAAGCCGCTCTGGAAGCTTTGCCTGATGATATGGATAAATTTATGGATGACTGTCTCACTAAATATAAATCAGAGGTGAAGGTTTTTAAACCGGAAAATTATGGTTTT**TAA**

**MtaC** (Uniprot ID: Q46EH4)

**ATG**GGTCATCATCACCATCACCACCACGGTTCTAGCGGGAGCTCGATGCTTGATTTCACCGAGGCCTCGCTGAAAAAGGTACTCACGCGTTACAACGTAGCATTAGAAAAAGCGCTTACGCCAGAGGAAGCAGCTGAAGAACTCTACCCCAAGGATGAGCTGATCTACCCCATTGCGAAAGCCATTTTTGAAGGAGAAGAAGATGATGTTGTCGAGGGACTTCAAGCTGCAATTGAGGCGGGGAAAGACCCGATCGATTTAATTGATGATGCGCTGATGGTTGGTATGGGGGTGGTGATCCGTCTGTATGATGAAGGAGTAATTTTTCTGCCTAATGTCATGATGTCCGCGGATGCAATGTTGGAAGGCATTGAATACTGCAAAGAAAACAGTGGTGCGACCCCTAAAACAAAAGGTACAGTCGTTTGCCATGTGGCCGAAGGGGATGTACATGACATCGGGAAAAACATTGTGACCGCCCTCTTACGCGCGAACGGGTATAATGTGGTGGACCTGGGACGTGATGTGCCAGCGGAAGAGGTGCTGGCAGCAGTGCAGAAAGAGAAACCGATCATGCTCACGGGTACCGCACTTATGACGACGACGATGTATGCCTTCAAAGAAGTTAATGATATGCTGTTGGAGAACGGGATTAAGATTCCGTTTGCTTGCGGCGGCGGCGCGGTGAACCAGGACTTCGTGAGCCAGTTCGCATTGGGCGTCTACGGTGAAGAAGCCGCAGATGCCCCAAAAATCGCGGATGCGATTATTGCAGGTACAACCGATGTTACGGAGTTACGGGAAAAATTTCATAAACAT**TAA.**

**MtgA** (Uniprot ID: Q24SP6)

**ATG**TTTAAGTTTACGGCCCAACAGCACGTATATGACATCAATGGTGTGAAGGTTGGCGGTCAGCCTGGCGAATATCCTACGGTTTTGATTGGAAGCATCTTTTACCGGGGTCACAAGATTGTTAGTGACGGTCAAAAGGGTATATTTGACAAGGACGCTGCTAAAGCGTTGCTCGACCAAGAGGCAGAGCTGTCAGCGGAAACGGGCAACCCTTTCATAATAGATGTTCTCGGCGAAAGCGTCGAGGCGCTCACTAAGTACGTAGAGTTTATCCTGGAGAACACCACAGCACCGTTTCTGCTCGATTCTATCTCCCCGGACGTTCGCGTTGGCGCGTTAAAGAATTTGGGTAAGGACCCGGAAATACAGAAACGGTTAATCTATAACTCGATAGAGGAACATTACACAGAAGAGGAACTCGCCGCAATCAAAGAGGCGGGCTTGAAGACGGCGGTCATACTGGCCTTTAGTAAGAAGGCCCTTAAACCCAATGCGCGTATAGATCTTTTGCAGGGGAAGGATGACAAGGAAGGCTTAATAGCAGCTGCGAAACGGGCGGGTATTGAGCAGTTCCTGGTAGACCCGGGCGTGCTGGACGTTGCCAGCAACTCCTGGACAACCGAGGCCATAAACGTCGTAAAGGAACAATTTGGTTACCCAGGTGGGTGTGCTCCGAGTAATGCAGTGTATCTTTGGAAGAAGATGCGCTCGAAAGGTACGCCCTTCTTCGAGGTGGCTGGAGCGGCTGTCTTCACCTATCCAATTACTCAGGGAGCGGACTTCATCCTTTACGGTCCTATGATGAATGCACCGTGGGTATATCGCGCAATCGCAACAACCGATGCTATGATTGCATACAATAACAAACTGACTGGCGTGAAGATGGGAACGACCGAGCACCCATTATTGAAGATATTC**TAA**

**MtgB (**Uniprot ID: Q24SP7)

**ATG**GGGCAACTGCTGCCTAAGTACAACATCCTGACAGAGGACCAGGTTCAAAAGATACACGAAAATACAATGAAAATTTTAGAAGAGATTGGTATCGAGTTCGAGTACGAGCCGGCACTTGAAGTGTTCCGTCGTGAGGGTCAAAAGGTGGAAGGTAAACGAGTATATTTAACCCGAGAGTTTGTGGAGTCCAAACTCAAGTCAGCACCGGCTGAATTCACGTTACACGCACGCAATCCTGAGAATAATGTTGTGATTGGTGGGGACAACATTGTCTTTATGCCTGGGTACGGCGCACCCTTCATTTATGAGTTAGACGGGAGCCGTCGTAAAACTACCTTACAAGACTACGAGAATTTTGCCAAACTGGCAGGCGCCAGTAAGAACATGCACTTATCTGGCGGCACGATGGCCGAACCGCAAGATATCCCAGACGGTGTGCGACATCTTCAAATGCTCTACTCCTCAATAAAGAACAGCGACAAGTGCTTTATGGGTTCCGCTGAAGGCAAGGAGAGAGCCGAGGATAGCGTTGAAATAGCAGCTATTCTGTTCGGTGGCAAGGACGTAATCAAAGAAAAGCCTGTTTTGGTATCCCTGATAAATTCTCTCACACCCCTTAAGTACGATGAGAGAATGTTAGGCGCATTGATGGCATACGCTGAGGCAGGCCAAGCCGTCATTATTGCAAGCCTGGTTATGGCCGGTTCGACAGGCCCCGCTAGTCTGGCTGGCACACTCTCATTACAAAACGCTGAAGTGTTGGCAGGTATAAGTCTGGCCCAATCCATTAATCCGGGCACCCCGGTAATATACGGTTCGACTAGTGCTCTTAGCGATATGCGCTCAGGCAGTTTGAGCATCGGAAGCCCTGAGTGTGCCCTTTTCATCTCCGCCAGCGCTCAGCTTGCACGGTTCTACGGTGTTCCCTCTCGCTCTGGCGGTGGGCTGAATGACTCGAAGACGGTGGACGCCCAAGCGGGGTACGAGAGTATGATGACCCTGATGGCAGCCAACCTCACCGGCGTTAACTTTGTGTTACACACCGCGGGGATTCTGCAGTATTTCATGGCGATGTCATACGAGAAGTTTATAATGGATGATGAGATCGCCGGGATGTTGTTACACTATATGAAGGGTTACACCTTTGACGAGGACGGTATGGCTTTCGACGTCATTGAGAAGGTGGGTCCTGGCGGTCATTTTCTGACGCAAAAGCACACTAGAAAGAACCATAAGCGCGAGTTTTACACCCCAACCTTATCAGACCGTTCGGCATATGACACGTGGGCGAAGGAGAAGTTGGAAACAAAGCAACGCGCACACGCGCGGTGGCAACAAATTTTGGCGAACTATGTTCCACCGGCTTTAGACCCTGAGATTGATGCCAAGCTGCAAGCGTTCATTGCCCAACGAGGCAAAGAGGTGGGCGAGGAA**TAA**

**MtgC (**Uniprot ID: Q24SP8)

**ATG**ATAATGTCTTTGTTAGACGAACTCAAGCAGGCAATCATTGATGGTGACGAGGACATAGTGGCTGAACTTTCGCAGAAGGCGGTTGACGAAGATCTTGATCTGGTTGACACCGTTCAATCGGGTCTCGTAAAGGGGATAGAGGTAGTTGGAACCGCTTGGAAAGAAGGGGAAATGTTTCTGCCAGATGTAATGATGTCAGCGGAAGCAATGAAAGTGGGCTTGGCCATTCTTGAACCAGAAATTGCAAAGAAAGGGATGAGTGAGGGTGAATCGAAAGGTAAGATTGTGCTTGGTACCGTTGAAGGTGATATCCATGATATTGGAAAGAATATCACTGGAGCAATGTTCACTGCCGCCGGATACAAAGTTATTGACTTAGGGACAGATATAAAGGCCGAGGGTTTTGTAGCCAAGGCCCAGGAAGTAGGCGCCGACATCATAGGCGCCTCAGCACTCCTCACCACCACGATGATTAAGCAAAAGGAGTTGATCGAGTATTTAAAGGAGAAGAACCTGCGAGACTCATATAAGGTACTCGTAGGTGGCGGGCCCACCTCTCAAGTTTGGGCGGACGAAATCGGAGCGGACGGATGGGCGGAGACAGCAGACGACGCCGTCGAGCTCGCAAATAAGATTTTGGGTAAG**TGA**

**M_1 (MtaB homolog, Uniprot ID: A0A0E3RY15)**

MAVNRCTKMSYASADEMIFGKAVKPVKAGLGLEIGAGYTTPEVNYAPRPEAGASKEKLIKEYERITTDIMARMVQIGAPAVVLETEHVQQMSNNPEWGAAVAHAQKTIMEDYHDEYGIKCALRHTIGDIRESRDFLELRGGDKYNTFLEAFEQCAKSGADMLSVESMGGKEIFDYAILRNDMAGILFGIGVLGSMDMEMIWKDISDIAKKTGTISAGDTDCAQANTAMFIAGGLLDKNLAHTIAIIARTISAARSLVAYEAGATGPGKDCGYENTIVKAIAGVPIAQEGKTSTCAHSDLMGNIVMQCCDLWSNESVEYHGEFGGTTVQCWSETLAYDCSMMNVALQSGNEKVLRDILVASDMYRDAQGYVLAYPHAYRVGEAIVRNGDDIYLRAKNAALETIKIIEEGAKGKLELSRFETTALANAKTSFEALTDDADKFMSDCLTKYKQEVKVFKPENYGL

**M_2 (MtaB homolog, Uniprot ID: A0A0E3SNR7)**

MTVTRYTKMAYANADDMVFGRAITPVKTGLELEIGAGYTAPEVNYAPRPEAGASKEKLIKEYERITTDIMARMVQIGAPAVVLETEHVQQMSNNPSWGAEVAHAQKTIMEDYHDEYGIKCALRHTIGDIRESREYLELRGGDKYNTFLEAFEQCAQNGADMLAVESMGGKEIFDYAILRNDMAGVLYGIGVLGSIDMEMIWQDIASIAKKTGTVASGDTDCAQANTAMFIAGGLLDKNLAHTLAIIARAISAARSLVAYEAGAVGPGKDCGYENTIVKSIAGVPITQEGKTSTCAHSDLMGNLVMQCCDLWSNESVEYHGEFGGTTVQCWSETLAYDCALMNVALESGNEKILRDMFMASDLYRDAQGYVLAYPNAYRVGQAIVKNGDDIYLRAKNAAIECINIVEEGAKKKLELSRFEAKALSDAKAAFEGLTDDKDQFMSDCLTKYKQEVKVFKPENYGL

**M_3 (MtaB homolog, Uniprot ID: A0A2A2HMT0)**

MAVTRCTKMAYANADDMVFGKAITPVKTGLGLEIGAGYTTPEVNYAPRPEAGASKEKLIKEYERITTDIMARMVQIGAPSVVLETEHVQQMSNNPDWGAAVAHAQKTIMEDYHDEYGIKCALRHTIGDIRESREYLELRGGDKYNTFLEAFEQCAQNGADMLAVESMGGKEVFDYAILRNDMAGVLYGIGVLGSIDMEMIWQDIASIAKKTGTVASGDTDCAQANTAMFIAGGLLDKNLAHTLAIIARAISAARSLVAYEAGAVGPGKDCGYENTIVKSIAGVPIAQEGKTSTCAHSDLMGNLVMQCCDLWSNESVEYHGEFGGTTVQCWSETLAYDCALMNVALKSGNEKILRDMFAASDLNRDAQGYVLAYPNAYRVGQAIVKNGDDIYLRAKNAAIECINIVEEGAKKKLELSRFEAKALSDAKAAFEGLTDDKDQFMSDCLTKYKQEVKVFKPENYGL

**M_4 (MtaB homolog, Uniprot ID: A0A1I7A7T7)**

MAVTRCTKMAYSSADEMVFGRAVNPVKAGLGLEIGAGYTTPEVNYAPRPEAGVSKEKLVKEYERITTDIMARMVQIGAPAVVLETEHVQQMSNNPDWGAEVAHAQKTIMEDYHDEYGIKCALRHTIGDIRESRDFLELRGDKYSVFMEAFEQCAENGADMLAVESMGGKEVFDYAILRNDMAGVLYGIGVLGSMDMEMIWQDIASIAKKNNVVASGDTDCAQANTAMFIAGGLLDKNLAHTLAIIARAVSAARSLVAYEAGAVGPGKDCGYENTICKAIAGVPISQEGKTSTCAHSDLMGNLTMQCCDLWSNESVEYHGEFGGTTVQCWAETLAYDCALMNVALQSGNEKILRDMFVASDMNRDAQGYVLAYNNAYRIGEAITRNGDDIYLRAKNAAIECINIIEEGAKKNLELSRFESKALSDAKAALESLTDDKEQFMSDCLTKYKQEIKVFKPENYGL

**M_5 (MtaB homolog, Uniprot ID: A0A7J4PNE7)**

MAANRCTKMAYASADDMIFGKSPKPIKAGLGLEIGAGYTTPEVNYAPRPEAGASKEKLIKEYERITTDIMARMVQIGAPAVVLETEHVQQMSNNPDWGAEVAHAQKAIMEDYHDEYGIKCALRHTIGDIRESRDFLQLRGDKYSVFMEAFEKCAENGADLLSVESMGGKEVFDYAILRNDMAGVLYGIGCLGGMDMEMIWSDIAAVAKKTGTVAAGDTDCAQANTAMFIAGGLLDKNLAHTLAIIARAISASRSLVAYEAGAVGPGKDCGYENTIVKSIAGVPIAQEGKTSTCAHSDVMGNLTMQCCDLWSNESVEYHGEFGGTTVQCWSETLAYDCALMNVALDSGNEKILRDMFAASDMYRDPQGYVLAYPNAYRVGQAIIKDGKDIYLRAKNAAIECINIVEEGARGKLALSRFESKSLADAKAAFEAMTDDQDKFMSDCLDKYKKEVKVFLPENYGL

B) **Mutagensis procedure**

Point mutations were introduced using overlap extension (SOE) PCR methods.^1^ The first step involved in the fragmentation PCR of the MtaA insert between NcoI and XhoI restriction sites using 1) a forward mutagenic primers with an insert-specific non-mutagenic reverse primers, and 2) reverse mutagenic primers with an insert-specific non-mutagenic forward primer. The fragmentation PCR products were treated with 1 unit DpnI in 30 µL of 1X NEB cutsmart buffer and purified by Qiagen PCR products purification kit. The assembly reactions were conducted using insert-specific non-mutagenic primers and the respective fragmentation PCR products. The assembled PCR products were purified by a 1% agarose gel and isolated using Qiagen gel purification kits. 1µg of the PCR products were digested by 1 unit of NcoI and 1 unit of XhoI enzymes in 50 µL of 1X NEB cutsmart buffer at 37 °C for 4 h, while 1µg of template plasmids were digested by the same conditions for 1h. The digested products were purified over 1% agarose gel and isolated using Qiagen gel purification kits. Ligations of the insert sequences into pET 28a+ were carried out by using 1 unit of T4 ligase with 1:10 molar ratio of digested PCR products and the double-digested pET28a (NcoI and XhoI) in 20 µL of 1X T4 ligation buffer at 22 °C for 3 h. The plasmids were purified by Qiagen PCR purification kits for transformations. Plasmid DNA was extracted from BL21 cells after transformation, and sequenced by ACGT (Chicago, IL) to confirm the results.

**Table S1.** Primer design for cloning different variants in MtaA using SOE-PCR.

| Primer Name | Sequence |
| --- | --- |
| Insert specific non-mutagenic F.P. | AGCATAACCCCTTGGGGCCTCTAAACG |
| Insert specific non-mutagenic R.P. | CGTTTAGAGGCCCCAAGGGGTTATGCT |
| MtaA T28G F.P | ATGCAGTGTCGGGCAGACCGGCATC |
| MtaA T28G R.P. | GATGCCGGTCTGCCCGACACTGCAT |
| MtaA Q29G F.P | ATGCAGTGTCACGGGGACCGGCATCGTA |
| MtaA Q29G R.P. | TACGATGCCGGTCCCCGTGACACTGCAT |
| MtaA R70G F.P. | GGAAGCGGTGGGGCTGCCATATTG |
| MtaA R70G R.P. | CAATATGGCAGCCCCACCGCTTCC |
| MtaA L75G F.P. | CTGCCATATTGTGGGACTGTATTAG |
| MtaA L75G R.P. | CTAATACAGTCCCACAATATGGCAG |
| MtaA Q94G F.P. | AATCGCGGGCCGAGCGTGACC |
| MtaA Q94G R.P. | GCTCGGCCCGCGATTTTTCGTA |
| MtaA A201G F.P. | TGTTATCGCCATCGGGGACCCTGTAGC |
| MtaA A201G R.P. | GCTACAGGGTCCCCGATGGCGATAACA |
| MtaA T28A F.P | ATGCAGTGTCGCGCAGACCGGCATC |
| MtaA T28A R.P. | GATGCCGGTCTGCGCGACACTGCAT |
| MtaA Q29A F.P | ATGCAGTGTCACGGCGACCGGCATCGTA |
| MtaA Q29A R.P. | TACGATGCCGGTCGCCGTGACACTGCAT |
| MtaA R70A F.P. | GGAAGCGGTGGCGCTGCCATATTG |
| MtaA R70A R.P. | CAATATGGCAGCGCCACCGCTTCC |
| MtaA L75A F.P. | CTGCCATATTGTGCGACTGTATTAG |
| MtaA L75A R.P. | CTAATACAGTCGCACAATATGGCAG |
| MtaA Q94A F.P. | AATCGCGCGCCGAGCGTGACC |
| MtaA Q94A R.P. | GCTCGGCGCGCGATTTTTCGTA |
| MtaA A201A F.P. | TGTTATCGCCATCGCGGACCCTGTAGC |
| MtaA A201A R.P. | GCTACAGGGTCCGCGATGGCGATAACA |

C) **Transformation**

Electro-competent BL21(DE3) *E. coli* cells were transformed by pET28a+ plasmids containing each enzyme insert between NcoI and XhoI restriction sites via electroporation and immediately recovered in 750 μL SOC medium. The cultures were incubated at 37 °C for 45 min. Then, the cells were spread on an LB agar plate containing 50 µg/mL kanamycin and incubated at 37 °C for 16 h. A single colony was used to inoculate 5mL LB media containing 50 µg/mL kanamycin, and the cells were cultured at 37 °C for 16 h. 1 mL of this culture was combined with 1 mL of 50% glycerol, and the resulting cell suspension was stored at -80 °C until further use. The recombinant plasmid containing the DNA template was extracted from overnight culture by QIAprep® miniprep kit with using protocols provided by the manufacturer and sequenced by Quintarabio.

D) **Protein Preparation**

7 mL overnight cultures were used to inoculate 1 L LB media containing 50 µg/mL antibiotic kanamycin (KAN). The cultures were incubated at 37 °C until OD_600_=0.6. Gene expression was induced by 1 mL of 1 mM IPTG at 16 °C for 18 h, and cells were harvested by centrifugation (3,200 g for 10 min). After the supernatant was discarded, the cells were resuspended into 10 mM imidazole buffer containing 50 mM sodium phosphate and 300 mM sodium chloride (pH=7.5) and stored at -80 °C until lysis. The cells were lysed by sonication at 4 °C for 5 min (30 s on/45 s off; 40 W). The crude extract was obtained after centrifugation at 31,000 x g and 4 °C for 20 min. The crude extract was mixed by end-over-end rotation with 5 mL Ni-NTA resin for 40 min and loaded into a gravity column. The repacked column was washed by 100 mL column wash buffer (20 mM imidazole, 50 mM sodium phosphate, and 300 mM sodium chloride, pH=7.5) to remove nonspecifically bound protein. The POI containing a His_6_ tag was eluted by using 60mL elution buffer containing 300 mM imidazole, 50 mM sodium phosphate and 300 mM sodium chloride (pH=7.5). The purified protein was dialyzed using 2x 4L 50 mM KP_i_ buffer at pH=7.5 overnight. After dialysis, the apoprotein was incubated with 3 equiv. MeCbl and buffer exchange was done to remove excess cofactor (note: size exclusion chromatography was not performed as MeCbl doesn’t show any background reactivity for methylation). Protein purity was evaluated by 12% SDS-PAGE electrophoresis as shown below in Figure S1-S2.

E) **MtaB renaturation procedure**

The cell pellet containing MtaB expressed from 0.5 L cell culture was resuspended in 30 mL of 50 mM MOPS/KOH pH 7.0. The cells were lysed by sonication at 4 °C for 5 min (30 s on/45 s off; 40 W). The crude extract was removed after centrifugation at 31,000 x g and 4 °C for 20 min. The resulting pellet containing MtaB as inclusion bodies was washed 4x15 mL of 50 mM Tris/HCl pH 8.0 containing 1 mM EDTA and 100 mM NaCl.^2^ Washed pellet was renatured at room temperature using 15 mL of 6.6 M urea and 10 mM dithiothreitol (DTT) in 50 mM MOPS/KOH pH 7.0 for 2h. Protein was refolded by performing 3-step dialysis^3^ (each step of dialysis had 66-fold less urea) in 50 mM MOPS/KOH pH 7.0 having 2 mM DTT. Dialysis was performed at 4L scale while slow stirring at 4 °C in anaerobic condition. After dialysis, insoluble protein was removed by centrifugation at 31,000 x g at 4 °C for 20 min. Soluble MtaB was further concentrated to 0.5 mM concentration using 10-kDa cut-off centrifugal spin filter column at 3600 rpm, 4 °C. Concentration MtaB was then directly used for catalysis without further purification.

**Table S2**. Summary of expression parameters for methyltransferase proteins in *E.coli.*

| Entry | Enzyme | Vector | Antibiotic  (50 µg/mL) | Cell growth (°C) | rpm | OD 600 | IPTG (mM), Temp (°C), t (h) |
| --- | --- | --- | --- | --- | --- | --- | --- |
| 1 | MtaA | pET28a(+) | KAN | 37 | 140 | 0.6-0.8 | 0.5, 25, 18 |
| 2 | MtaB | pET16b | Amp | 37 | 250 | 0.5 | 0.1, 18, 18, 1 mM ZnSO_4_ |
| 3 | MtaC | pET28a(+) | KAN | 37 | 250 | 0.5 | 0.5, 18, 18 |
| 4 | MtgA | pET28a(+) | KAN | 37 | 250 | 0.6-0.8 | 1.0, 37, 4 |
| 5 | MtgB | pET28a(+) | KAN | 37 | 250 | 0.6-0.8 | 1.0, 37, 4 |
| 6 | MtgC | pET28a(+) | KAN | 37 | 250 | 0.6-0.8 | 1.0, 37, 4 |

A)

B)

C)

**Figure S1.** (A) 12% SDS-PAGE showing purification for MTases using Ni-NTA affinity chromatography. Marker (M, lane 1), blank (lane 2 and 7) and 1,3,5 and 6 mg/mL (lane 3,4,5 and 6 from left) of MtaA (39 kDa) and 1,3,5 and 6 mg/mL (lane 8,9,10 and 11) of MtgB (54 kDa) protein obtained after Ni-NTA affinity purification. (B) 1,3, and 5 mg/mL of MtgA (35 kDa) in lane 2,3 and 4 and 1,3 and 5 mg/mL of MtaC (22 kDa) in lane 6,7 and 8. (C) Lane S3 (0.5 mg/mL), S5 (1.0 mg/mL) and S10 (1.5 mg/mL) as soluble MtaB (54 kDa) after renaturation and lane P3, P5 and P10 as insoluble MtaB after renaturation.

F) **MtaA glycine/alanine variants protein preparation**

The same procedure as described in section II.D was followed for expression and purification of glycine and alanine mutants for six active site residues (Q29, T28, L75, A201, Q94, R70) of MtaA.

**Figure S2.** 12% SDS-PAGE showing purified elution fractions: 1(5 mg/ml) & 2(2 mg/mL): Q29G, 3 & 4: R70G, 5 & 6: L75G, 7 & 8: A201G, 9 & 10: T28G lysate, 11: purified T28G, 12 & 13: Q94G lysate, 14: purified Q94G

G) **ESI-MS spectrum for purified His-tagged proteins**

Protein molecular weight determination by ESI-MS: The molecular masses of purified proteins were determined from the multiply charged electrospray ionization mass spectra by deconvolution of the charge-state envelope. Charge states (z) were assigned from adjacent peaks using the relationship z=(m2−H)/(m2−m1), where m1and m2 are the m/z values of adjacent charge states and H is the mass of a proton (1.007276 Da). The neutral molecular mass (M) was then calculated for each assigned charge state according to M=z(m/z−H). The calculated molecular masses obtained from multiple charge states were averaged to obtain the final deconvoluted molecular mass of each protein (note: the baseline is not visible in the deconvoluted spectra due a software error).

Raw ESI-MS spectrum for MtaA (39511 Da):

Deconvoluted ESI-MS spectrum for purified MtaA (39511 Da):

Raw ESI-MS spectrum for purified MtaB (54245 Da):

Deconvoluted ESI-MS spectrum for purified MtaB (54245 Da):

Raw ESI-MS spectrum for purified MtaC (22851 Da):

Deconvoluted ESI-MS spectrum for purified MtaC (22851 Da):

Raw ESI-MS spectrum for purified MtgA (35124 Da):

Deconvoluted ESI-MS spectrum for purified MtgA (35124 Da):

Raw ESI-MS spectrum for purified MtgB (54240 Da):

Deconvoluted ESI-MS spectrum for purified MtgB (54240 Da):

III. **AF-3 analysis of engineered variant Q29G**

AF-3 docking of **5b** with MtaA variant Q29G:

**L75**

**G29**

**5b**

AF-3 docking of **5b** with parent MtaA:

**Q29**

**5b**

Active-site pocket volume analysis: To evaluate the structural consequences of the Q29G substitution on the substrate-binding pocket, cavity volumes were calculated using the DoGSiteScorer module available through the ProteinsPlus Structure-Based Modeling Support Server.^4^ The highest-ranked pocket, corresponding to the substrate-binding site identified from the AlphaFold3 docking models, was used for comparison between the parent MtaA and the Q29G variant. The calculated pocket volume increased from 492.67 Å^3^ for the parent enzyme to 608 Å^3^ for the Q29G variant, indicating expansion of the active-site cavity upon replacement of Gln29 with Gly.

**Parent MtaA**

(492 Å^3^)

**Q29G**

(608 Å^3^)

IV. **Docking analysis of stereoselectivity**

**AF3 docking analysis of substrate positioning**: To gain more insight into the origin of the observed enantioselectivity, AF3 docking studies were performed using the prochiral substrate **5a** in the MtaA+MtaC system. The predicted binding mode was first compared with the available crystal structure of MtaA (PDB: 4AY8) in complex with its native substrate, coenzyme M (CoM). Notably, the binding orientation of **5a** closely resembles the observed binding pose of CoM, with thiol group of **5a** occupying the similar catalytic position as the thiol group of the native substrate adjacent to the methylcobalamin cofactor. This orientation positions the substrate in a geometry consistent with productive methyl transfer while preserving the key substrate recognition features observed for the native enzyme-substrate complex.

***pro*-S**

***pro*-R**

**Figure S3.** AF3 docking models of the prochiral substrate **5a** in the MtaA–MtaC complex containing the zinc ion and methylcobalamin cofactor overlayed with crystal structure of MtaA complexed with native substrate CoM (PDB ID: 4AY8), showing the predominant binding orientation (*pro*-R) and the alternative binding orientation (*pro*-S).

To assess whether alternative binding modes could account for formation of the opposite enantiomer, twenty independent AF3 docking models were generated. 13 of the 20 models, including all top ranked models (Figure S4), converged to the *pro*-R binding orientation and positions the enantiotopic thiol for methyl transfer to produce R enantiomer. In contrast, seven model adopted the opposite binding orientation (*pro*-S), which would be expected to generate the opposite enantiomer. The preference of independently generated AF3 models toward *pro*-R binding orientation is consistent with the observed stereochemistry.

***pro*-R**

***pro*-S**

**Figure S4.** Overlay of twenty independently generated AF3 docking models of the prochiral substrate **5a** in the MtaA+MtaC complex containing the zinc ion and methylcobalamin cofactor, displayed as two subsets in form of *pro*-R and *pro*-S binding orientation of **5a**. Thirteen models including top ranked models in each run adopted the *pro*-R binding orientation, whereas seven model adopted the *pro*-S binding orientation.

V. **X-ray crystallographic determination of the absolute configuration of the 6a**

Procedure: A semipreparative-scale bioconversion was carried out in a total reaction volume of 30 mL (divided in 24 X 1.5 mL eppendorf) containing **5a** (10 mM, 50 mg), GB (2.0 equiv), MtgB (0.0125 mol%), MtaA/C (0.025 mol%), Ti(III) citrate (4 mM), and ZnCl_2_ (40 µM) in 50 mM MOPS/KOH buffer (pH 7.0). The reaction mixture was stirred at 750 rpm and 37 °C in thermomixer under N_2_ for 24 h, after which quantitative conversion was confirmed by LC-MS. The reaction was quenched by the addition of 0.1 M HCl, and the precipitated enzymes were removed by filtration through Celite. The filtrate was neutralized with aqueous NaHCO_3_, extracted into ethyl acetate, and concentrated under reduced pressure to afford crude **6a** in upto 80 mL of ethyl acetate. The crude product containing **6a** was then treated with trimethylsilyldiazomethane (2.0 equiv) to afford the corresponding methyl ester derivative **6b**, which was purified by using a Biotage system equipped with a C18 column using 0-5% H_2_O/MeCN gradient as the eluent to give **6b** in 51% isolated yield. Purified **6b** was subsequently dissolved in DMF and treated with *p*-bromobenzyl bromide (1.0 equiv) in the presence of Cs_2_CO_3_ (1.2 equiv) at 0 °C for 1 h to afford **6d** in 31% isolated yield after purification by silica gel column chromatography (95:5 hexanes/ethyl acetate). Compound **6d** was dissolved in DCM and was then oxidized with *m*-CPBA (4.12 equiv) at room temperature overnight to produce sulfone **6e** in 56% isolated yield following purification by silica gel column chromatography using 5-10% MeOH in CH_2_Cl_2_ as the eluent.

**X-Ray	characterization:**

**Crystallization**

Single crystals suitable for X-ray diffraction were obtained by carefully layering pentane over a dichloromethane solution of the compound **6e**. The sample was left undisturbed at room temperature, affording crystals suitable for X-ray diffraction.

**Sample information**

A colorless, needle-shaped specimen of C_13_H_17_BrO_6_S_2_, approximate dimensions 0.02×0.02×0.13 mm^3^, was placed on a Kapton mount with inert oil for crystal structure determination. The X-ray intensity data were measured on a Bruker D8 Venture KAPPA diffractometer equipped with a microfocus sealed tube (λ = 0.71073 Å) and a multilayer mirror monochromator. The experiment was performed at 153(2) K using an Oxford Cryostream open nitrogen-flow temperature control unit.

**Data Collection**

The data collection was performed using 1.0° ω and φ scans, frame times of 20 s, and a detector distance of 50 mm. with a total exposure time of 4.5 hours. The frames were integrated with the SAINT V8.41 package using a narrow-frame algorithm. [1] The integration of the data using a monoclinic unit cell yielded 18063 reflections to a maximum θ angle of 26.40° (0.80 Å resolution), of which 3274 were independent (average redundancy 5.52, completeness = 99.9%, R_int_ = 5.06%, R_sig_ = 3.62%) and 3061 (93.5%) were greater than 2σ(*F*^2^). The final cell constants of a = 5.3242(10) Å, b = 8.8309(13) Å, c = 17.117(3) Å, α = 90°, β = 95.162(6)°, γ = 90°, volume = 801.5(2) Å^3^, are based upon the refinement of the XYZ-centroids of 5763 reflections above 20 σ(I) with 2.39° < 2θ < 25.88°. Data were corrected for absorption effects using the Multi-Scan method in SADABS 2016/2. The calculated minimum and maximum transmission coefficients (based on crystal size) are 0.708 and 0.945.

**Structure solution and refinement**

The space group *P*2_1_ (#4) was determined based on intensity statistics and systematic absences. The structure was solved by SHELXT 2018/2 and refined with full-matrix least squares / difference. Fourier cycles using SHELXL-2019/2; Z = 2 for the formula unit C_13_H_17_BrO_6_S_2_. [3,4]. Non-hydrogen atoms were refined with anisotropic displacement parameters. The hydrogen atoms were placed in ideal positions and refined as riding atoms with relative isotropic displacement parameters. The final anisotropic full-matrix least-squares refinement on *F*^2^ with 201 variables against 3274 data points and 1 restraint converged at *R*_1_ = 2.68%, for the observed data and wR_2_ = 5.96% for all data. The goodness-of-fit on *F*^2^ was 1.04. The largest peak in the final difference electron density synthesis was 0.25 e−/Å^3^ and the deepest hole was −0.21 e−/Å^3^ with an RMS deviation of 0.058 e−/Å^3^. On the basis of the final model, the calculated density was 1.71 g/cm^3^ and *F*(000), 420 e−.

**Refinement Details**

The enantiomer of the molecule present in the crystal is the S enantiomer. The absolute configuration of the crystal and molecule was established via XRD, with a refined Flack X parameter of -0.001(4). The priority of the sulfur-containing substituents is reversed in the reaction product (CO_2_^-^>CH_2_SMe>CH_2_SH), indicating that the R enantiomer of the product is formed in the enzymatic methylation reaction.

X-Ray structure of **6e** (C_13_H_17_BrO_6_S_2_):

VI. **Bioconversion Protocol**

**A) Analytical scale reaction procedure**

Analytical scale bioconversion (400 $\mu$L): Thiol/aniline methylation bioconversions were set up in a glovebox under N_2_ atmosphere using a 1.5 mL plastic Eppendorf tube. Bioconversion reactions were set up by adding thiol/aniline and glycine betaine (GB) or MeOH in 50 mM MOPS/KOH, pH 7.0 into the reaction vial, followed by the addition of catalyst (preincubated with reducing agent for 1 min) under the anaerobic condition at 37 °C. The final concentration of each reagent was: 10 mM Thiol or Aniline, 20 mM GB or 5% v/v MeOH, 0.5 mol% MtgB or 5 mol% MtaB, 1 mol% MtaA or MtgA, 1 mol% MtaC, 4 mM Ti(III) citrate. After the reaction setup, the reaction vials were agitated by shaking on 24 well plate shaker at 750 rpm for overnight at 37 °C. After completion of the bioconversion, the reactions were quenched by the precipitation of enzyme using 0.1M HCl solution and addition of 400 µL of ACN. The reaction mixture was collected in 2 mL Eppendorf tube and further centrifuged to separate the enzyme residues. Crude reaction mixtures were filtered through a 20 μM filter, analyzed by LC-MS, and product conversion were determined either by area integration using extracted ion chromatograms (EICs) or by isolating products.

**B) Semipreparative scale reaction**

Semi-preparative scale bioconversion (25 mL): Biotransformations on a semi-preparative scale were set up in a glove box under N_2_ atmosphere using a 100 mL round bottom flask charged with a magnetic stir bar. Thiol substrates, for example **1c**, **1e** or **1f** (50 mg), and 2 equivalents of GB were dissolved in 10% v/v MeOH in 50 mM MOPS/KOH, pH 7.0, were added to the flask. For the scale-up bioconversion, 1.0 mol% of MtaC preincubated with Ti(III) citrate (final concentration 4 mM), 0.5 mol% of MtgB and 1.0 mol% of MtaA were added to the reaction mixture. The flask was then sealed with a septum and kept for stirring at 750 rpm overnight at 37 °C. After overnight stirring, quantitative conversions were observed by LC-MS. The crude reaction mixture was quenched by the addition of 0.1 M hydrochloric acid. The precipitated enzyme was removed through celite filtration. The supernatant was then neutralized by sodium bicarbonate solution. The organic layer was then extracted and concentrated under reduced pressure. After purification by automated flash column chromatography (9.5:0.5 hexane/EtOAc), the product was obtained in a 93% yield (65 mg). Same semi-preparative scale procedure was followed for the **5a** and **5b** substrate, and the products were isolated in 52% and 40% yield respectively using flash column chromatography using C18 column (9.9:0.1 H_2_O/ACN).

VII. **Native activity measurement and assay development**

A) **Methylation of OHCbl catalyzed by MtxB enzyme**

**Table S3.** Conversion for methylation of OHCbl using glycine betaine (10 mM) or MeOH (5% v/v) as methyl donor catalyzed by MtxB proteins and control reactions without MtxB proteins. Conversion is based on UV-Vis area integration at 280 nm of MeCbl vs OHCbl.

| *Entry* | *B* | *MtxB* | *%conv.* |
| --- | --- | --- | --- |
| 1 | GB | MtgB | 50% |
| 2 | MeOH | MtaB | 30% |
| 3 | GB | No catalyst | 0% |
| 4 | MeOH | No catalyst | 0% |

**Authentic standards**

**Figure S6.** Chromatogram showing formation of MeCbl catalyzed by MtaB protein.

B) **Methylation of native substrates catalyzed by MtxA enzymes**

**Table S4.** Conversion for methylation of native substrate CoM or THF using stochiometric amount of MeCbl as methyl donor catalyzed by MtxA proteins and control reactions without MtxA proteins. Conversion is based on ratio of area integration of product over sum of area of substrate and product using LC-MS ESI negative mode.

| *Entry* | *A* | *MtxA* | | | *%conv.* | |
| --- | --- | --- | --- | --- | --- | --- |
| 1 | CoM | | | MtaA | >99% | |
| 2 | THF | | | MtgA | >99% | |
| 3 | CoM | | No catalyst | | 0% |  |
| 4 | THF | | | No catalyst | 0% | |

VIII. **Reaction optimization for bioconversion using MeOH and glycine betaine**

**Ti(III) Citrate concentration optimization**

% Conv. to 2a

| Ti(III) Citrate (mM) | % Conv. (LCMS) |
| --- | --- |
| 1 | 25 |
| 2 | 28 |
| 4 | 23 |
| 8 | 20 |
| 10 | 15 |

**Figure S7**. Conversion for CoM methylation using different concentrations of Ti(III) Citrate catalyzed by MtaABC.

**ZnCl_2_ concentration optimization**

| ZnCl2 (mM) | % conv. to 2a |
| --- | --- |
| 0.5 | 25 |
| 1 | 32 |
| 2 | 35 |
| 5 | 20 |
| 10 | 8 |

**Figure S8**. Conversion for CoM methylation using different concentrations of ZnCl_2_ catalyzed by MtaABC.

**%MeOH (v/v) optimization**

| % MeOH (v/v) | % Conv. to 2a |
| --- | --- |
| 2 | 30 |
| 5 | 37 |
| 10 | 32 |
| 15 | 30 |
| 20 | 20 |

**Figure S9**. Conversion for CoM methylation using different concentrations of methanol catalyzed by MtaABC.

**Reaction buffer optimization**

% Conv. to 2a

| pH | HEPES | MES | PIPES | MOPS |
| --- | --- | --- | --- | --- |
| 6.5 | 15 | 25 | 25 | 20 |
| 7 | 18 | 35 | 38 | 33 |
| 7.5 | 29 | 36 | 35 | 37 |
| 8 | 23 | 35 | 34 | 32 |

**Figure S10**. Conversion for CoM methylation across different buffer conditions catalyzed by MtaABC.

**Time course**

| Time (h) | % Conv. to 2a |
| --- | --- |
| 0 | 0 |
| 0.5 | 25 |
| 1 | 30 |
| 2 | 32 |
| 4 | 35 |
| 6 | 36 |
| 12 | 35 |
| 22 | 38 |

**Figure S11**. Conversion for CoM methylation at different time points catalyzed by MtaABC.

**Effect of addition of different enzymes after 10 h**

| Rxn Time (h) | % Conv.to 2a |
| --- | --- |
| 5 1 | 39 |
| 5 2 | 38 |
| 5 3 | 37 |
| 10 1+MtaA | 38 |
| 10 2+MtaB | 40 |
| 10 3+MtaC | 37 |

**Figure S12**. Conversion for CoM methylation at different time points with addition of extra equivalent of each enzyme.

**MtaB catalyst loading optimization**

| MtaB (mol%) | % Conv. to 2a |
| --- | --- |
| 0.5 | 15 |
| 1 | 25 |
| 2 | 35 |
| 5 | 43 |
| 10 | 38 |
| 20 | 40 |

**Figure S13**. Conversion for CoM methylation at different MtaB loading.

**GB concentration optimization**

| GB conc. (mM) | % Conv. to 2e |
| --- | --- |
| 10 | 75 |
| 20 | 100 |
| 40 | 100 |
| 50 | 100 |

**Figure S14**. Conversion for methyl-3-mercaptopropionate methylation at different concentration of glycine betaine catalyzed by MtgB-MtaA/C enzymes.

**Co-solvents optimization for 5b desymmetrization**

| Cosolvent | % Conversion (6b) |
| --- | --- |
| MeOH | 15 |
| ACN | 11 |
| none | 6 |
| IPA | 2 |
| DMF | 2 |
| DMSO | 0.9 |
| THF | 0.5 |

**Figure S15**. Reaction scheme and conversion for **5b** methylation using different co-solvents catalyzed by MtgB+MtaA(Q29G)+MtaC enzymes.

IX. **Reaction optimization and TON for monomethylation**

**Table S5.** Time course and different catalyst loadings to optimize formation of monomethylated product **6a** and dimethylated product **6a’** for dithiol substrate **5a**.

| Entry | Time  (h) | %  Catalyst loading | MtgB (uM) | MtaC (uM) | MtaA (uM) | MeCbl (uM) | % 6a (m/z 165) | % 6a’ (m/z 179) | % Conv.  overall^a^ | | Turnover number | |
| --- | --- | --- | --- | --- | --- | --- | --- | --- | --- | --- | --- | --- |
| 1 | 0 | 0.05 | 5 | 12.5 | 12.5 | 15.5 | 0 | 0 | | 0 | | 0 |
| 2 | 0.5 | 0.05 | 5 | 12.5 | 12.5 | 15.5 | 17 | 0 | | 17 | | 340 |
| 3 | 1.0 | 0.05 | 5 | 12.5 | 12.5 | 15.5 | 22 | 0 | | 22 | | 440 |
| 4 | 2.0 | 0.05 | 5 | 12.5 | 12.5 | 15.5 | 29 | 0 | | 29 | | 580 |
| 5 | 4.0 | 0.05 | 5 | 12.5 | 12.5 | 15.5 | 61 | 8 | | 69 | | 1380 |
| 6 | 6.0 | 0.05 | 5 | 12.5 | 12.5 | 15.5 | 52 | 25 | | 77 | | 1540 |
| 7 | 9.0 | 0.05 | 5 | 12.5 | 12.5 | 15.5 | 73 | 20 | | 93 | | 1860 |
| 8 | 23.0 | 0.05 | 5 | 12.5 | 12.5 | 15.5 | 41 | 59 | | 99 | | 1980 |
| 9 | 0 | 0.025 | 2.5 | 6.25 | 6.25 | 7.5 | 0 | 0 | | 0 | | 0 |
| 10 | 0.5 | 0.025 | 2.5 | 6.25 | 6.25 | 7.5 | 4.6 | 0 | | 4.6 | | 184 |
| 11 | 1.0 | 0.025 | 2.5 | 6.25 | 6.25 | 7.5 | 10.0 | 0 | | 10.0 | | 400 |
| 12 | 2.0 | 0.025 | 2.5 | 6.25 | 6.25 | 7.5 | 8 | 0 | | 8.0 | | 320 |
| 13 | 4.0 | 0.025 | 2.5 | 6.25 | 6.25 | 7.5 | 28.4 | 0 | | 28.4 | | 1136 |
| 14 | 6.0 | 0.025 | 2.5 | 6.25 | 6.25 | 7.5 | 37.5 | 1.3 | | 39 | | 1560 |
| 15 | 9.0 | 0.025 | 2.5 | 6.25 | 6.25 | 7.5 | 52 | 2 | | 54 | | 2160 |
| 16 | 23.0 | 0.025 | 2.5 | 6.25 | 6.25 | 7.5 | 62 | 21 | | 83 | | 3320 |
| 17 | 0 | 0.0125 | 1.25 | 3.125 | 3.125 | 3.75 | 0 | 0 | | 0 | | 0 |
| 18 | 0.5 | 0.0125 | 1.25 | 3.125 | 3.125 | 3.75 | 1.2 | 0 | | 1.2 | | 96 |
| 19 | 1.0 | 0.0125 | 1.25 | 3.125 | 3.125 | 3.75 | 2.9 | 0 | | 2.9 | | 232 |
| 20 | 2.0 | 0.0125 | 1.25 | 3.125 | 3.125 | 3.75 | 4.3 | 0 | | 4.3 | | 344 |
| 21 | 4.0 | 0.0125 | 1.25 | 3.125 | 3.125 | 3.75 | 16 | 0 | | 16 | | 1280 |
| 22 | 6.0 | 0.0125 | 1.25 | 3.125 | 3.125 | 3.75 | 24 | 0 | | 24 | | 1920 |
| 23 | 9.0 | 0.0125 | 1.25 | 3.125 | 3.125 | 3.75 | 26 | 0 | | 26 | | 2080 |
| 24 | 23.0 | 0.0125 | 1.25 | 3.125 | 3.125 | 3.75 | 54 | 3 | | 57 | | 4560 |
| 25 | 25.0 | 0.0125 | 1.25 | 3.125 | 3.125 | 3.75 | 72 | 4 | | 76 | | 6080 |

^a^conversion is based on ratio of area of products over sum of area of remaining substrate and products based on ESI (negative mode).

X. **Michaelis-Menten kinetics of the desymmetrization of 5a**

Procedure: Analytical scale bioconversion (400 $\mu$L) using **5a** were set up in triplicate in a glovebox under N_2_ atmosphere using a 1.5 mL plastic Eppendorf tube. The final concentration of each reagent was: substrate **5a** (ranging from 0 mM, 0.2 mM, 0.5 mM, 1 mM, 1.2 mM, 1.5 mM, 2 mM, 4 mM, 6 mM and 8 mM), 2 eq. GB, 0.0125 mol% MtgB, 0.025 mol% MtaA/C and 4 mM Ti(III) citrate under the anaerobic condition at 37 °C. After the reaction setup, the reaction vials were agitated by shaking on 24 well plate shaker at 750 rpm at 37 °C. 50 µL aliquot of reactions at different time points (ranging from 0 h, 1 h, 2 h, 3 h, 5 h, and 6 h) were quenched by the precipitation of enzyme by addition of 50 µL of ACN containing 2 mM TMB as internal standard. The reaction mixture was collected in 2 mL Eppendorf tube and further centrifuged to separate the enzyme residues. Crude reaction mixtures were filtered through a 20 μM filter, analyzed by LC-MS, and product concentration were determined by calibration curve of **6a** product standard using area integration from extracted ion chromatograms (EICs) compared to TMB as internal standard.

Initial rate (µM/min)

| 5a (mM) | Experimental data |
| --- | --- |
| 0 | 0 |
| 0.2 | 0.49984 |
| 0.5 | 1.03 |
| 1 | 1.38903 |
| 1.2 | 1.57366 |
| 1.5 | 1.81306 |
| 2 | 2.13333 |
| 4 | 2.32201 |
| 6 | 2.38936 |
| 8 | 2.48714 |

| 5a (mM) | Michaelis Menten Fit |
| --- | --- |
| 0 | 0 |
| 0.0808081 | 0.22765 |
| 0.161616 | 0.420546 |
| 0.242424 | 0.586084 |
| 0.323232 | 0.729697 |
| 0.40404 | 0.855471 |
| 0.484848 | 0.966535 |
| 0.565657 | 1.06533 |
| 0.646465 | 1.15378 |
| 0.727273 | 1.23343 |
| 0.808081 | 1.30552 |
| 0.888889 | 1.3711 |
| 0.969697 | 1.431 |
| 1.05051 | 1.48592 |
| 1.13131 | 1.53647 |
| 1.21212 | 1.58315 |
| 1.29293 | 1.62638 |
| 1.37374 | 1.66654 |
| 1.45455 | 1.70393 |
| 1.53535 | 1.73884 |
| 1.61616 | 1.77151 |
| 1.69697 | 1.80214 |
| 1.77778 | 1.83092 |
| 1.85859 | 1.85801 |
| 1.93939 | 1.88356 |
| 2.0202 | 1.90769 |
| 2.10101 | 1.93052 |
| 2.18182 | 1.95216 |
| 2.26263 | 1.97269 |
| 2.34343 | 1.99219 |
| 2.42424 | 2.01074 |
| 2.50505 | 2.02842 |
| 2.58586 | 2.04527 |
| 2.66667 | 2.06136 |
| 2.74747 | 2.07673 |
| 2.82828 | 2.09144 |
| 2.90909 | 2.10552 |
| 2.9899 | 2.11902 |
| 3.07071 | 2.13197 |
| 3.15152 | 2.1444 |
| 3.23232 | 2.15634 |
| 3.31313 | 2.16783 |
| 3.39394 | 2.17888 |
| 3.47475 | 2.18953 |
| 3.55556 | 2.19979 |
| 3.63636 | 2.20968 |
| 3.71717 | 2.21923 |
| 3.79798 | 2.22844 |
| 3.87879 | 2.23735 |
| 3.9596 | 2.24596 |
| 4.0404 | 2.25429 |
| 4.12121 | 2.26235 |
| 4.20202 | 2.27015 |
| 4.28283 | 2.27771 |
| 4.36364 | 2.28504 |
| 4.44444 | 2.29215 |
| 4.52525 | 2.29904 |
| 4.60606 | 2.30573 |
| 4.68687 | 2.31223 |
| 4.76768 | 2.31854 |
| 4.84848 | 2.32468 |
| 4.92929 | 2.33064 |
| 5.0101 | 2.33645 |
| 5.09091 | 2.34209 |
| 5.17172 | 2.34759 |
| 5.25253 | 2.35294 |
| 5.33333 | 2.35815 |
| 5.41414 | 2.36323 |
| 5.49495 | 2.36818 |
| 5.57576 | 2.373 |
| 5.65657 | 2.37771 |
| 5.73737 | 2.3823 |
| 5.81818 | 2.38679 |
| 5.89899 | 2.39116 |
| 5.9798 | 2.39544 |
| 6.06061 | 2.39961 |
| 6.14141 | 2.40369 |
| 6.22222 | 2.40767 |
| 6.30303 | 2.41157 |
| 6.38384 | 2.41538 |
| 6.46465 | 2.4191 |
| 6.54545 | 2.42275 |
| 6.62626 | 2.42631 |
| 6.70707 | 2.4298 |
| 6.78788 | 2.43322 |
| 6.86869 | 2.43657 |
| 6.94949 | 2.43984 |
| 7.0303 | 2.44305 |
| 7.11111 | 2.4462 |
| 7.19192 | 2.44928 |
| 7.27273 | 2.4523 |
| 7.35354 | 2.45527 |
| 7.43434 | 2.45817 |
| 7.51515 | 2.46102 |
| 7.59596 | 2.46382 |
| 7.67677 | 2.46656 |
| 7.75758 | 2.46925 |
| 7.83838 | 2.47189 |
| 7.91919 | 2.47448 |
| 8 | 2.47703 |

Vmax: 2.754 ± 0.01 µM/min, K_M_: 0.89 ± 0.02 mM, kcat: 2.2 ± 0.01 min^-1^.

**Figure S16**. Michaelis–Menten plot for the desymmetrization of **5a** catalyzed by the MtgB+MtaA/C methyltransferase system. Initial reaction rates were determined at varying substrate concentrations under the optimized reaction conditions and fitted to the Michaelis–Menten equation to obtain the kinetic parameters.

XI. **Turnover number measurement for thiol methylation**

**Table S6.** Reaction optimization to isolate product **2e** having high turnover number.

| Entry | µM MtaA/C | Mol% (X) | | % Conv. | | TON |  |
| --- | --- | --- | --- | --- | --- | --- | --- |
| 1 | 25 | | 0.25 | | >99 | 396 | |
| 2 | 12.5 | | 0.125 | | >99 | 792 | |
| 3 | 6.25 | | 0.062 | | >99 | 1584 | |
| 4 | 3.125 | | 0.031 | | 83 | 2656 | |
| 5 | 1.56 | | 0.015 | | 29 | 1859 | |
| 6 | 0.78 | | 0.0078 | | 13 | 2080 | |
| 7 | 0.40 | | 0.0040 | | 3 | 962 | |
| 8 | 0.20 | | 0.0020 | | trace | n.d. | |

XII. **Methylation with MeI to measure site selectivity of mono vs demethylation**

**Table S11.** Conversion to **2c** using stoichiometric MeI as methyl donor.

| Entry | MeI  (mM) | Na_2_CO_3_ (eq.) | | %Conv. (2c) | | Mono vs di ratio | |
| --- | --- | --- | --- | --- | --- | --- | --- |
| 1 | 10 | 0 | 0 | | 0 | |  |
| 2 | 20 | 0 | 0 | | 0 | |  |
| 3 | 10 | 1.2 | >99 | | 50:50 | |  |
| 4 | 20 | 1.2 | >99 | | 1:100 | |  |

EIC chromatogram for formation of 50:50 mixture of mono and dimethylated product when methylation reaction was carried out using 1 eq. of MeI as shown in entry 3, Table S11.

EIC chromatogram for formation of 1:100 mixture of mono and dimethylated product when methylation reaction was carried out using 2 eq. of MeI as shown in entry 4, Table S11.

XIII. **Stoichiometric methylation**

**Table S12.** Conversion for methylation of non-native thiols and anilines using stoichiometric MeCbl as methyl donor. 10% MeOH as a co-solvent was added for entry 4-10 for better solubility.

| Entry | Substrate | Catalyst (5 mol%) | | Conversion | Method |
| --- | --- | --- | --- | --- | --- |
|  |  | MtaA | MtaC |  |  |
| 1 |  | $\boldsymbol{✓}$ | $\boldsymbol{🗶}$ | >99 % | LC-MS std. |
|  |  | $\boldsymbol{🗶}$ | $\boldsymbol{✓}$ | >99 % | LC-MS std. |
| 2 |  | $\boldsymbol{✓}$ | $\boldsymbol{🗶}$ | >99 % | LC-MS std. |
|  |  | $\boldsymbol{🗶}$ | $\boldsymbol{✓}$ | No conv. | LC-MS std. |
| 3 |  | $\boldsymbol{✓}$ | $\boldsymbol{🗶}$ | trace | GC-MS conv. |
|  |  | $\boldsymbol{🗶}$ | $\boldsymbol{✓}$ | trace | GC-MS conv. |
|  |  | $\boldsymbol{✓}$ | $\boldsymbol{✓}$ | 40 % | GC-MS conv. |
|  |  | $\boldsymbol{🗶}$ | $\boldsymbol{✓}$(DhaF4611) | trace | GC-MS conv. |
|  |  | $\boldsymbol{✓}$ | $\boldsymbol{✓}$(DhaF4611) | 90 % | GC-MS conv. |
| 4 |  | $\boldsymbol{✓}$ | $\boldsymbol{🗶}$ | trace | GC-MS conv. |
|  |  | $\boldsymbol{🗶}$ | $\boldsymbol{✓}$ | trace | GC-MS conv. |
|  |  | $\boldsymbol{✓}$ | $\boldsymbol{✓}$ | 60 % | GC-MS conv. |
|  |  | $\boldsymbol{✓}$ | $\boldsymbol{✓}$(DhaF4611) | trace | GC-MS conv. |
| 5 |  | $\boldsymbol{✓}$ | $\boldsymbol{🗶}$ | trace | GC-MS conv. |
|  |  | $\boldsymbol{🗶}$ | $\boldsymbol{✓}$ | trace | GC-MS conv. |
|  |  | $\boldsymbol{✓}$ | $\boldsymbol{✓}$ | trace | GC-MS conv. |
| 6 |  | $\boldsymbol{✓}$ | $\boldsymbol{🗶}$ | 10 % | LC-MS conv. |
|  |  | $\boldsymbol{🗶}$ | $\boldsymbol{✓}$ | 5 % | LC-MS conv. |
|  |  | $\boldsymbol{✓}$ | $\boldsymbol{✓}$ | 12% | LC-MS conv. |
| 7 | Tetrahydrofolate | $\boldsymbol{✓}$ (MtgA) | $\boldsymbol{🗶}$ | >99 % | LC-MS std. |
| 8 |  | $\boldsymbol{✓}$ | $\boldsymbol{🗶}$ | No conv. | LC-MS conv. |
|  |  | $\boldsymbol{🗶}$ | $\boldsymbol{✓}$ | No conv. | LC-MS conv. |
|  |  | $\boldsymbol{✓}$ | $\boldsymbol{✓}$ | No conv. | LC-MS conv. |
|  |  | $\boldsymbol{✓}$ (MtgA) | $\boldsymbol{✓}$ | No conv. | LC-MS conv. |
| 9 |  | $\boldsymbol{✓}$ (MtgA) | $\boldsymbol{🗶}$ | trace | LC-MS conv. |
|  |  | $\boldsymbol{✓}$ (MtgA) | $\boldsymbol{✓}$ | trace | LC-MS conv. |
| 10 |  | $\boldsymbol{✓}$ (MtgA) | $\boldsymbol{🗶}$ | trace | LC-MS conv. |
|  |  | $\boldsymbol{✓}$ (MtgA) | $\boldsymbol{✓}$ | trace | LC-MS conv. |

**Table S13.** Conversion for stoichiometric thiol methylation catalyzed by MtaA and MtaC together. 10% MeOH as a co-solvent was added for entry 4-10 for better solubility.

| Entry | Substrate | Conversion | Method |
| --- | --- | --- | --- |
| 1 |  | 10 % | LC-MS conv. |
| 2 |  | 80 % | GC-MS conv. |
| 3 |  | >99 % | GC-MS conv. |
| 4 |  | >99 % | LC-MS conv. |
| 5 |  | 30 % | LC-MS conv. |
| 6 |  | No conv. | GC-MS conv. |
| 7 |  | trace | LC-MS conv. |
| 8 |  | No conv. | GC-MS conv. |
| 9 |  | No conv. | GC-MS conv. |
| 10 |  | 15 % | GC-MS conv. |

XIV. **Bioconversion analysis for thiol and aniline substrates**

**UPLC-MS Method**

Column specifications: Agilent Eclipse Plus C18 column (3.5 μm particle size; 4.6 x 150 mm). Eluents used as water with 0.1% formic acid as mobile phase A, and ACN with 0.1% formic acid as mobile phase B having flow rate of 0.4 mL/min.

**Table S14.** Following table shows UPLC-MS method for analyzing the methylated products.

| Time (min) | %A | %B |
| --- | --- | --- |
| 0 | 100 | 0 |
| 1.5 | 100 | 0 |
| 3 | 70 | 30 |
| 5 | 0 | 100 |
| 5 | 0 | 100 |
| 5.5 | 100 | 0 |

LC-MS analysis for bioconversions was performed on an Agilent 1200 series system connected to a ZORBAX Eclipse Plus C18 column (2.1 mm x 50 mm, 1.8 μm particle size) with a 1290 Infinity II high-speed pump, a 1260 Infinity II diode array detector, and a 1290 Infinity II multisampler with single-needle configuration connected to a 6135X single quadrupole mass spectrometer with an Agilent Jet Stream ESI source. Yields for **2a**, **2b**, **2d**, **2g**, **2i**, **4a**, and **6c**, were determined by LC-MS or GC-MS relative to internal standard using response factors derived from authentic standards (see section XII below). Yields for **2c**, **2e**, **2f**, **6a**, and **6b**, were determined by product isolation from semi-preparative scale bioconversions. Conversions to **2h, 2j-l**, and **4b-4d** were determined by LC-MS extracted ion chromatograms (EICs) obtained at the m/z of the substrate and product from each reaction as provided in Table S15. Conversions to **2k** was determined by GC-MS using peak integrals from the GC-MS chromatogram and the expression given above. Conversion (%) is defined as (product area/(product area + substrate area))*100 for triplicate measurements.

**Table S15.** Method used for reported product yields and conversions.

| Compound | Substrate *m/z* | Product *m/z* | Ion Species | Method |
| --- | --- | --- | --- | --- |
| 2a | 141 | 155 | [M-H]^-^ | LC-MS, Std. |
| 2b | 155 | 169 | [M-H]^-^ | LC-MS, Std. |
| 2c | 187 | 201 | [M-H]^-^ | LC-MS, Conv., Isol. |
| 2d | 136 | 150 | [M+H]^+^ | LC-MS, Conv; Std. |
| 2e | n/a | n/a | n/a | GC-MS, Conv., Isol. |
| 2f | n/a | n/a | n/a | GC-MS, Conv., Isol. |
| 2g | 190 | 204 | [M+H]^+^ | LC-MS, Conv., Std. |
| 2h | 170 | 184 | [M+H]^+^ | LC-MS, Conv. |
| 2i | 141 | 155 | [M+H]^+^ | LC-MS, Conv., Std. |
| 2j | 152 | 166 | [M+H]^+^ | LC-MS, Conv. |
| 2k | 192 | 206 | [M]^+^ | GC-MS, Conv. |
| 2l | 181 | 195 | [M+H]^+^ | LC-MS, Conv. |
| 4a | 446 | 460 | [M+H]^+^ | LC-MS, Conv., Std. |
| 4b | 196 | 210 | [M+2H]^+^ | LC-MS, Conv. |
| 4c | 240 | 254 | [M+H]^+^ | LC-MS, Conv. |
| 4d | 162 | 176 | [M+H]^+^ | LC-MS, Conv. |
| 6a | n/a | n/a | n/a | LC-MS, Conv., Isol. |
| 6b | n/a | n/a | n/a | GC-MS, Conv., Isol. |
| 6c | 180 | 194 | M^+^ | GC-MS, Conv., Std. |

Pdt

SM

**Figure S17**. EIC for reaction of **1a** to give **2a** with GB as methyl donor (no SM remained).

SM

Pdt

**Figure S18**. EIC for reaction of **1a** to give **2a** with MeOH as methyl donor.

Pdt

SM

**Figure S19**. EIC for control reaction for **1a** without enzyme MtxABC (no product formed).

Pdt

**Figure S20**. EIC for reaction of **1b** to give **2b** with GB as methyl donor (no SM remained).

Pdt

**Figure S21**. EIC for reaction of **1c** to give **2c** with GB as methyl donor (no SM remained).

**Figure S22**. Mass spectrum trace showing formation of monomethylation vs dimethylation for **1c.**

Pdt

**Figure S23**. EIC for reaction of **1d** to give **2d** using GB as methyl donor (no SM remained).

Pdt

Pdt **2e** MS trace

**Figure S24**. GC chromatogram and MS trace for reaction of **1e** to give **2e** using GB as methyl donor (no SM remained).

SM

Pdt

**Figure S25**. GCMS trace for reaction **1e** to give **2e** using MeOH as methyl donor.

Pdt

SM

**Figure S26**. EIC for reaction of **1g** to give **2g** using GB as methyl donor.

SM

Pdt

**Figure S27**. EIC for reaction of **1h** to give **2h** using GB as methyl donor.

SM

Pdt

**Figure S28**. EIC for reaction of **1i** to give **2i** using GB as methyl donor.

SM

Pdt

**Figure S29**. EIC for reaction of **1j** to give **2j** using GB as methyl donor.

SM

Pdt

**Figure S30**. EIC for reaction of **1l** to give **2l** using GB as methyl donor.

Pdt

SM

**Figure S31.** EIC for reaction of **3a** to give **4a** using GB as methyl donor.

Pdt

SM

**Figure S32.** Control reaction for **3a** methylation without MtgB-MtaAC enzyme.

SM

Pdt

**Figure S33.** EIC for reaction of **3b** to give **4b** using GB as methyl donor.

SM

No Pdt

**Figure S34.** EIC for reaction **3b** without MtgAB-MtaC catalyst.

SM

Pdt

**Figure S35.** EIC for reaction of **3c** to give **4c** using GB as methyl donor.

Pdt

SM

**Figure S36.** EIC for reaction of **3d** to give **4d** using GB as methyl donor.

**Figure S37.** UHPLC chromatogram for reaction of **5a** to give **6a**. E1 and E2 are enantiomer for racemic product standard.

Monomethylated pdt **6b**

Dimethylated pdt **6b’**

SM

Pdt **6b** MS trace

**Figure S38.** GC-MS chromatogram for reaction of **5b** to give **6b** and **6b’** using GB as methyl donor.

MS trace of pdt **6c**

**Figure S39.** GC-MS for reaction of **5c** to give **6c** using GB as methyl donor.

XV. **LCMS Calibration Curves**

The calibration curves were constructed by plotting the EICs integration ratios of 800 nmol of internal standard TMB and EICs of authentic products **2a**, **2b**, **2d**, **2g**, **2i**, **4a**, and **6c** in 800 µL of acetonitrile solution. Each sample was prepared by mixing 480 nmol of internal standard with 400-4000 nmol of authentic products in 800 µL of acetonitrile solution. All the samples were analyzed by LC-MS instruments.

| 2a (mM) | EIC area (2a/TMB) |
| --- | --- |
| 1 | 0.224988 |
| 2 | 0.765872 |
| 4 | 1.58769 |
| 6 | 2.31022 |
| 8 | 2.90275 |
| 10 | 3.96463 |

**Figure S40.** Calibration curve of product **2a**. Representative data points were constructed by adding 480 nmol of Internal Standard (TMB) with 400-4000 nmol of authentic **2a** in 800 µL of acetonitrile solution.

| 2b (mM) | EIC area (2b/TMB) |
| --- | --- |
| 1 | 0.301736 |
| 2 | 0.650242 |
| 4 | 1.06979 |
| 5 | 1.32226 |
| 10 | 2.32475 |

**Figure S41.** Calibration curve of product **2b**. Representative data points were constructed by adding 480 nmol of Internal Standard (TMB) with 400-4000 nmol of authentic **2b** in 800 µL of acetonitrile solution.

| 2d (mM) | EIC area (2d/TMB) |
| --- | --- |
| 1 | 0.675805 |
| 2 | 2.2762 |
| 4 | 4.64276 |
| 6 | 6.8472 |
| 8 | 8.74827 |
| 10 | 11.657 |

**Figure S42.** Calibration curve of product **2d**. Representative data points were constructed by adding 480 nmol of Internal Standard (TMB) with 400-4000 nmol of authentic **2d** in 800 µL of acetonitrile solution.

| 2g (mM) | EIC area (2g/TMB) |
| --- | --- |
| 1 | 0.438726 |
| 2 | 0.651786 |
| 4 | 1.03512 |
| 5 | 1.40953 |

**Figure S43.** Calibration curve of product **2g**. Representative data points were constructed by adding 480 nmol of Internal Standard (TMB) with 400-2000 nmol of authentic **2g** in 800 µL of acetonitrile solution.

| 2i (mM) | EIC area (2i/TMB) |
| --- | --- |
| 1 | 0.591706 |
| 2 | 1.36673 |
| 4 | 2.32831 |
| 5 | 2.71842 |
| 10 | 4.58055 |

**Figure S44.** Calibration curve of product **2i**. Representative data points were constructed by adding 480 nmol of Internal Standard (TMB) with 400-4000 nmol of authentic **2i** in 800 µL of acetonitrile solution.

| 4a (mM) | EIC area (4a/TMB) |
| --- | --- |
| 1 | 0.314399 |
| 2 | 1.13886 |
| 4 | 2.34634 |
| 6 | 3.58942 |
| 8 | 4.43742 |
| 10 | 5.80468 |

**Figure S45.** Calibration curve of product **4a**. Representative data points were constructed by adding 480 nmol of Internal Standard (TMB) with 400-4000 nmol of authentic **4a** in 800 µL of acetonitrile solution.

| 6a (mM) | EIC Area (6a/TMB) |
| --- | --- |
| 0.05 | 0.0318916 |
| 0.1 | 0.0461751 |
| 0.2 | 0.057576 |
| 0.4 | 0.0835336 |
| 0.8 | 0.137581 |
| 1 | 0.150323 |
| 2 | 0.306417 |

**Figure S46.** Calibration curve of product **6a**. Representative data points were constructed by adding 480 nmol of Internal Standard (TMB) with 400-2000 nmol of authentic **6a** in 800 µL of acetonitrile solution.

| 6c (mM) | GC-MS area (6c/TMB) |
| --- | --- |
| 1 | 0.583353 |
| 2 | 1.29341 |
| 4 | 2.53569 |
| 5 | 3.23246 |

**Figure S47.** Calibration curve of product **6c**. Representative data points were constructed by adding 480 nmol of Internal Standard (TMB) with 400-2000 nmol of authentic **6c** in 800 µL of acetonitrile solution.

XVI. **Chiral chromatogram**

**A)** Desymmetrization of **5a**

Method: Diacel Chiralpak OJ-3 column, UHPLC-1290, 98Hex-2IPA isocratic, 3ml/min.

**B)** Desymmetrization of **5b**

Method: Diacel Chiralpak IC-3, UHPLC, 1% IPA, 99% Hexane, 2 ml/min.

Enzymatic reaction

Racemic product **6b**

**C)** Desymmetrization of **5c**

Method: Diacel Chiralpak IC-3, UHPLC, 0.5% n-propanol, 99.5% Hexane, 2 ml/min.

XVII. **Synthetic Procedures**

A. **Synthesis of product standards 2b, 2g, and 2i:**

**Procedure for synthesis of 2b**^5^: Thiol **1b** (1 mmol) was dissolved in MeOH and treated with NaOH (1.0 equiv.), followed by dropwise addition of methyl iodide (1.0 equiv.) under nitrogen at room temperature. The reaction mixture was stirred overnight, quenched with diethyl ether (50 mL), and the resulting white precipitate was collected by filtration, washed with diethyl ether (20 mL X 3), and dried under vacuum to afford the desired product **2b** in 50% yield.

**^1^H NMR** (500 MHz, CD_3_OD) 2.93 – 2.89 (m, 2H), 2.64 – 2.61 (m, 2H), 2.08 (s, J = 1.0 Hz, 3H), 2.08 – 2.02 (m, 2H).

**^13^C NMR** (126 MHz, CD_3_OD) δ 49.94, 32.42, 24.17, 13.60. LCMS (ESI) calcd. for [C_4_H_9_O_3_S_2_]^−^ [M−H]^−^: 169.0, found: 169.1.

**Procedure for synthesis of 2g**: Thiol **1g** (0.5 mmol) was treated with methyl iodide (1.0 equiv.) and triethylamine (2.0 equiv.) in MeOH under nitrogen atmosphere at room temperature and stirred overnight, which gave full conversion confirmed by LC-MS, after which the crude reaction mixture was purified by silica gel column chromatography to obtain desired product **2g** in 45% yield.

**^1^H NMR** (500 MHz, CD_3_OD) 3.77 (s, 2H), 2.67 (s, 3H), 2.30 (s, 3H).

**^13^C NMR** (126 MHz, CD_3_OD) δ 176.11, 168.85, 153.45, 127.46, 34.91, 19.50, 17.23. LCMS (ESI) calcd. for [C_7_H_10_NO_2_S_2_]^+^ [M+H]^+^: 204.0, found: 204.1.

**Procedure for synthesis of 2i**: Thiol **1i** (0.5 mmol) was treated with methyl iodide (0.8 equiv.) and Cs_2_CO_3_ (1.0 equiv.) in EtOH under nitrogen at room temperature for 3 h, and the product was purified by reverse-phase flash chromatography on a C18 column using ACN/H_2_O (0.5:9.5, isocratic) to afford the desired product **2i** in 30% yield.

**^1^H NMR** (500 MHz, CDCl_3_) 8.47 – 8.42 (m, 2H), 8.38 (s, 1H), 3.05 (t, J = 7.4 Hz, 2H), 2.86 (dd, J = 7.9, 6.9 Hz, 2H), 2.06 (s, 3H).

**^13^C NMR** (126 MHz, CDCl_3_) δ 144.76, 144.18, 142.59, 35.05, 33.30, 15.68. LCMS (ESI) calcd. for [C_7_H_11_N_2_S]^+^ [M+H]^+^: 155.1, found: 155.1.

B. **Enzymatic synthesis of N-benzylmaleimide derivative of 2c**

**Procedure:** Biotransformations on a semi-preparative scale were set up in a glove box under N_2_ atmosphere using a 100 mL round bottom flask charged with a magnetic stir bar. Thiol substrates, for example **1c** (20 mg), and 2 equivalents of GB were dissolved in 50 mM MOPS/KOH, pH 7.0, were added to the flask. 0.05 mol% of MtaC preincubated with Ti(III) citrate (final concentration 4 mM), 0.025 mol% of MtgB and 0.05 mol% of MtaA were added to the reaction mixture. The flask was then sealed with a septum and kept for stirring at 750 rpm overnight at 37 °C. After overnight stirring, quantitative conversions were observed by LC-MS. The crude reaction mixture was quenched by the addition of 50 mL of ACN and stirred for 2 hours at 37 °C in air. The precipitated enzyme was removed through celite filtration. 2equiv. of N-benzylmaleimide was then added to the supernatant and reaction was monitored using LC-MS for complete conversion to **2c’**. Crude reaction mixture was then treated with concentrated HCl to reduce the pH to 1. The organic layer was then extracted using ethyl acetate (50 ml X 5). The combined organic layers were concentrated under reduced pressure, and the crude product was purified by flash chromatography (C18, 50 g column; eluent: H_2_O/ACN with 0.1% formic acid, 99.5:0.5). Fraction containing pure 2c’ were then combined and frozen using liquid nitrogen for lyophilization to afford the N-benzylmaleimide derivative **2c’** in 40% yield.

**^1^H NMR** (500 MHz, CD_3_OD) 7.42 – 7.12 (m, 5H), 4.66 (s, 2H), 4.20 – 4.07 (m, 1H), 3.55 (dd, J = 13.5, 4.0 Hz, 1H), 3.37 (d, J = 12.4 Hz, 1H), 3.31 – 3.26 (m, 1H), 3.25 – 3.17 (m, 2H), 3.11 (ddd, J = 17.6, 13.9, 3.4 Hz, 1H), 2.67 – 2.53 (m, 1H), 2.15 (s, 3H).

**^13^C NMR** (126 MHz, CD_3_OD) δ 181.10, 179.38, 132.16, 131.71, 131.66, 131.29, 58.35, 58.19, 47.13, 46.47, 45.78, 45.73, 44.34, 43.61, 39.83, 39.80, 38.76, 38.30, 16.24, 16.21. LCMS (ESI) calcd. for [C_15_H_18_NO_5_S_3_]^−^ [M−H]^−^: 388.0, found: 388.0.

^1^H NMR of **2c’**:

^13^C NMR of **2c’**:

HMBC:

The HMBC spectrum showed a correlation between the methyl protons and the adjacent carbon next to sulfur at 43.2 ppm. The HSQC spectrum revealed that the protons attached to this carbon displayed only one correlation with another carbon, supporting the formation of desired product **2c’**. In contrast, structure **2c’’** was excluded because Ha’ would be expected to show two HSQC correlations, which were not observed.

HSQC

LC-MS ESI trace for purified N-benzylmaleimide product derivative **2c’**:

C. **Synthesis of methyl and ester derivatives 5b-c**

**General procedure**^6^: A solution of **5a** (1.0 mmol, 1.0 equiv) in MeOH or **5a** (1.0 mmol, 1.0 equiv) in EtOH (10 mL) was treated with concentrated H_2_SO_4_ (2–3 drops) and heated to reflux for 20 h. The reaction mixture was then cooled to room temperature and extracted with ethyl acetate. The combined organic layers were concentrated under reduced pressure, and the crude residue was purified by column chromatography on silica gel (hexane/ethyl acetate, 9.5:0.5) to afford the corresponding ester products **5b** and **5c** in 60% and 90% yield respectively.

**^1^H NMR** (600 MHz, CDCl_3_) δ 3.75 (s, 3H), 2.92 (ddd, *J* = 13.5, 8.7, 6.6 Hz, 2H), 2.87 – 2.84 (m, 1H), 2.85 – 2.76 (m, 2H), 1.47 (t, *J* = 8.6 Hz, 2H).

**^13^C NMR** (126 MHz, CDCl_3_) δ 172.64, 52.11, 51.19, 24.53. GCMS calcd. for [C_5_H_10_O_2_S_2_]^+^ [M]^+^: 166.0, found: 166.1.

**^1^H NMR** (500 MHz, CDCl_3_) δ 4.21 (q, *J* = 7.1 Hz, 2H), 2.92 (ddd, *J* = 13.6, 8.6, 6.6 Hz, 2H), 2.89 – 2.86 (m, 1H), 2.86 – 2.72 (m, 2H), 1.47 (t, *J* = 8.6 Hz, 2H), 1.30 (t, *J* = 7.1, 0.7 Hz, 3H).

**^13^C NMR** (126 MHz, CDCl_3_) δ 172.10, 61.05, 51.17, 24.54, 14.23. GCMS calcd. for [C_6_H_12_O_2_S_2_]^+^ [M]^+^: 180.0, found: 180.1.

D. **Synthesis of monomethyl racemic product 6a-c**

**Procedure for synthesis of 6a:** To a stirred solution of dithiol substrate **5a** (1.0 mmol) in MeOH (10 mL) was added K_2_CO_3_ (2.0 equiv) under a N_2_ atmosphere at room temperature, methyl iodide (0.2 eq.) dissolved in 1mL methanol was added dropwise, and the reaction mixture was stirred at room temperature overnight. The reaction was quenched, and the mixture was extracted with ethyl acetate. The combined organic layers were concentrated under reduced pressure, and the crude product was purified by HPLC-C18 column using a gradient method of 20-33% ACN-H_2_O, for 20 min, followed by 33:67 ACN-H_2_O for 10 min at flow rate of 2 ml/min. Fraction containing desired product were combined and extracted with ethyl acetate and concentrated under reduced pressure to afford desired racemic product **6a** in 2% yield.

**^1^H NMR** (500 MHz, CDCl_3_) 2.85 (ddd, *J* = 13.6, 8.6, 6.6 Hz, 2H), 2.82 – 2.79 (m, 1H), 2.79 – 2.72 (m, 2H), 2.08 (s, 3H), 1.50 (t, 1H).

**^13^C NMR** (126 MHz, CDCl_3_) δ 178.10, 47.92, 34.27, 24.69, 16.03. LCMS (ESI) calcd. for [C_5_H_10_O_2_S_2_]^-^ [M-H]^-^: 165.0, found: 165.1.

**Procedure for synthesis of 6b**: To a stirred solution of dithiol substrate **5b** (0.5 mmol) in MeOH (5 mL) was added K_2_CO_3_ (1.0 equiv) under a N_2_ atmosphere at room temperature, methyl iodide (0.2 equiv.) dissolved in 1mL methanol was added dropwise, and the reaction mixture was stirred at room temperature overnight. The reaction mixture was concentrated under reduced pressure, and crude product mixture was redissolved in ACN and 1eq. of Ti(III) Citrate was added and left for stirring for 12 h. The reaction was quenched, and the mixture was extracted with ethyl acetate. The combined organic layers were concentrated under reduced pressure, and the crude product was purified by HPLC-C18 column using isocratic method having 50:50 ACN-H_2_O for 30 min at flow rate of 3 ml/min. Fraction containing desired product were combined and extracted with ethyl acetate and concentrated under reduced pressure to afford desired racemic product **6b** in 2.5% yield.

**^1^H NMR** (500 MHz, CDCl_3_) 3.68 (s, 3H), 2.80 (ddd, *J* = 13.5, 8.4, 5.8 Hz, 2H), 2.78 – 2.77 (m, 1H), 2.72 (m, 2H), 2.05 (s, 3H), 1.44 (t, *J* = 8.3 Hz, 1H).

**^13^C NMR** (126 MHz, CDCl_3_) δ 173.20, 52.06, 48.28, 34.70, 25.07, 15.92. GCMS calcd. for [C_6_H_12_O_2_S_2_]^+^ [M]^+^: 180.0, found: 180.1.

**Procedure for synthesis of 6c**: To a stirred solution of dithiol substrate **5c** (0.233 mmol) in EtOH (5 mL) was added Cs_2_CO_3_ (1.0 equiv) under a N_2_ atmosphere at room temperature, methyl iodide (0.2 equiv) dissolved in 1mL ethanol was added dropwise, and the reaction mixture was stirred at room temperature for 1h. The reaction was quenched, and the mixture was extracted with DCM. The combined organic layers were concentrated under reduced pressure, and the crude product was purified by HPLC-C18 column using 50:50 ACN-H_2_O for 30 min at flow rate of 3 ml/min. Fraction containing desired product were combined and extracted with ethyl acetate and concentrated under reduced pressure to afford desired product **6c** in 7% yield.

**^1^H NMR** (500 MHz, CDCl_3_) 4.23 – 4.10 (m, 2H), 2.85 – 2.78 (m, 2H), 2.78 – 2.75 (m, 1H), 2.75 – 2.67 (m, 2H), 2.06 (s, 3H), 1.43 (t, J = 8.6 Hz, 1H), 1.23 (t, J = 7.1 Hz, 3H).

**^13^C NMR** (126 MHz, CDCl_3_) δ 172.79, 61.09, 48.29, 34.95, 25.10, 15.94, 14.25. GCMS calcd. for [C_7_H_14_O_2_S_2_]^+^ [M]^+^: 194.0, found: 194.1.

^1^H NMR of **2b**

^13^C NMR of **2b**

^1^H NMR of **2g**

^13^C NMR of **2g**

^1^H NMR of **2i**

H-grease

^13^C NMR of **2i**

^1^H NMR of **5b**

^13^C NMR of **5b**

^1^H NMR of **5c**

^13^C NMR of **5c**

^1^H NMR of **6c**

^13^C NMR of **6c**

**NMR for isolated products using semi-preparative enzymatic bioconversion**

^1^H NMR of **2e**

^13^C NMR of **2e**

^1^H NMR of **2f**

^13^C NMR of **2f**

^1^H NMR of **6a**

^13^C NMR of **6a**

^1^H NMR of **6b**

^13^C NMR of **6b**

^1^H NMR of **6e**

^13^C NMR of **6e**

XVIII. **References**

(1)	Heckman, K. L.; Pease, L. R. Gene Splicing and Mutagenesis by PCR-Driven Overlap Extension. *Nat. Protoc.* **2007**, *2* (4), 924–932. https://doi.org/10.1038/nprot.2007.132.

(2)	Sauer, K.; Thauer, R. K. Methanol:Coenzyme M Methyltransferase from Methanosarcina Barkeri. Zinc Dependence and Thermodynamics of the Methanol:Cob(I)Alamin Methyltransferase Reaction. *Eur. J. Biochem.* **1997**, *249* (1), 280–285. https://doi.org/10.1111/j.1432-1033.1997.t01-1-00280.x.

(3)	Yamaguchi, H.; Miyazaki, M. Refolding Techniques for Recovering Biologically Active Recombinant Proteins from Inclusion Bodies. *Biomolecules*. MDPI AG March 1, 2014, pp 235–251. https://doi.org/10.3390/biom4010235.

(4)	Volkamer, A.; Kuhn, D.; Rippmann, F.; Rarey, M. DoGSiteScorer: A Web Server for Automatic Binding Site Prediction, Analysis and Druggability Assessment. *Bioinformatics* **2012**, *28* (15), 2074–2075. https://doi.org/10.1093/bioinformatics/bts310.

(5)	Wang, L.; Carrow, B. P. Oligothiophene Synthesis by a General C–H Activation Mechanism: Electrophilic Concerted Metalation–Deprotonation (ECMD). *ACS Catal.* **2019**, *9* (8), 6821–6836. https://doi.org/10.1021/acscatal.9b01195.

(6)	Curran, D.; Dada, O.; Müller-Bunz, H.; Rothemund, M.; Sánchez-Sanz, G.; Schobert, R.; Zhu, X.; Tacke, M. Synthesis and Cytotoxicity Studies of Novel NHC∗-Gold(I) Complexes Derived from Lepidiline A. *Molecules* **2018**, *23* (8). https://doi.org/10.3390/molecules23082031.
